# Novel Semisynthetic Derivatives of Bile Acids as Effective Tyrosyl-DNA Phosphodiesterase 1 Inhibitors

**DOI:** 10.3390/molecules23030679

**Published:** 2018-03-17

**Authors:** Oksana V. Salomatina, Irina I. Popadyuk, Alexandra L. Zakharenko, Olga D. Zakharova, Dmitriy S. Fadeev, Nina I. Komarova, Jóhannes Reynisson, H. John Arabshahi, Raina Chand, Konstantin P. Volcho, Nariman F. Salakhutdinov, Olga I. Lavrik

**Affiliations:** 1 N.N. Vorozhtsov Novosibirsk Institute of Organic Chemistry, SB RAS, acad. Lavrentjev ave. 9, Novosibirsk 630090, Russia; ana@nioch.nsc.ru (O.V.S.); popadyuk@nioch.nsc.ru (I.I.P.); dsf@nioch.nsc.ru (D.S.F.); komar@nioch.nsc.ru (N.I.K); anvar@nioch.nsc.ru (N.F.S.); 2 Novosibirsk Institute of Chemical Biology and Fundamental Medicine, SB RAS, acad. Lavrentjev ave. 8, Novosibirsk 630090, Russia; sashaz@niboch.nsc.ru (A.L.Z); isar@niboch.nsc.ru (O.D.Z.); lavrik@niboch.nsc.ru (O.I.L.); 3 School of Chemical Sciences, University of Auckland, Auckland 1142, New Zealand; j.reynisson@auckland.ac.nz (J.R.); j.arabshahi@auckland.ac.nz (H.J.A.); rcha387@aucklanduni.ac.nz (R.C.); 4 Novosibirsk State University, Pirogova str. 2, Novosibirsk 630090, Russia

**Keywords:** deoxycholic acid, chenodeoxycholic acid, ursodeoxycholic acid, amide, Tdp1 inhibitor, cancer, tumor, virtual screening, molecular modelling

## Abstract

An Important task in the treatment of oncological and neurodegenerative diseases is the search for new inhibitors of DNA repair system enzymes. Tyrosyl-DNA phosphodiesterase 1 (Tdp1) is one of the DNA repair system enzymes involved in the removal of DNA damages caused by topoisomerase I inhibitors. Thus, reducing the activity of Tdp1 can increase the effectiveness of currently used anticancer drugs. We describe here a new class of semisynthetic small molecule Tdp1 inhibitors based on the bile acid scaffold that were originally identified by virtual screening. The influence of functional groups of bile acids (hydroxy and acetoxy groups in the steroid framework and amide fragment in the side chain) on inhibitory activity was investigated. In vitro studies demonstrate the ability of the semisynthetic derivatives to effectively inhibit Tdp1 with IC_50_ up to 0.29 µM. Furthermore, an excellent fit is realized for the ligands when docked into the active site of the Tdp1 enzyme.

## 1. Introduction

Systems that are responsible for repairing DNA damage play a crucial role in maintaining genome integrity. However, the increased activity of such systems produces significant problems in the treatment of a variety of immune, neurodegenerative diseases and, especially, cancerous tumors [1,2,3]. The cytotoxic effects of chemo- and radiotherapy are often based on DNA damage and these methods are used in clinical practices that treat malignant tumors. The ability of tumor cells to recognize and repair DNA damage can lead to a resistance to certain anti-cancer medications. Thus, the inhibition of DNA repair enzymes can increase the effectiveness of several types of antitumor drugs that are in clinical use [4]. Therefore, the search for inhibitors of the DNA repair system enzymes is one of the most crucial research areas in medical chemistry today [5,6,7].

Tyrosyl-DNA phosphodiesterase 1 (Tdp1) plays an important role in removing DNA damage caused by topoisomerase 1 (Top1) inhibitors [3,8,9]. Tdp1 catalyzes the hydrolysis of adducts covalently bound to DNA’s 3′-phosphate, including Top1 peptides, Top1–inhibitor complexes, as well as 3′-phosphoglycolates and others [2,10,11]. In addition, Tdp1 is capable of cleaving 5′-adducts of DNA [12]. As a result, Tdp1 counteracts not only drugs aimed at inhibiting Top1 (camptothecins, indenoisoquinolines), but also topoisomerase II (Top2) inhibitors (etoposide, doxorubicin), bleomycin and DNA alkylating agents. It is suggested that namely Tdp1 is responsible for the drug resistance of some cancers [2,6]. A number of studies supports this hypothesis. For example, it is known that mice Tdp1 knock-out models are hypersensitive to camptothecin [13,14,15,16]. Conversely, in cells with elevated levels of Tdp1 expression, camptothecin and etoposide cause less DNA damage [17,18]. It has also been shown that the suppression of Tdp1 activity makes tumor cells hypersensitive to anticancer drugs with other mechanisms of action: temozolomide (purine methylation) [19], methyl methanesulfonate (formation of apurine/apyrimidine sites), bleomycin (single-stranded/double-stranded breaks with 3′-phosphoglycolates), hydrogen peroxide and ionizing radiation (breaks and other types of damage) [20]. Thus, Tdp1 is considered a promising target enzyme in the development of drugs for the treatment of oncological diseases [2,21].

**Figure 1:**
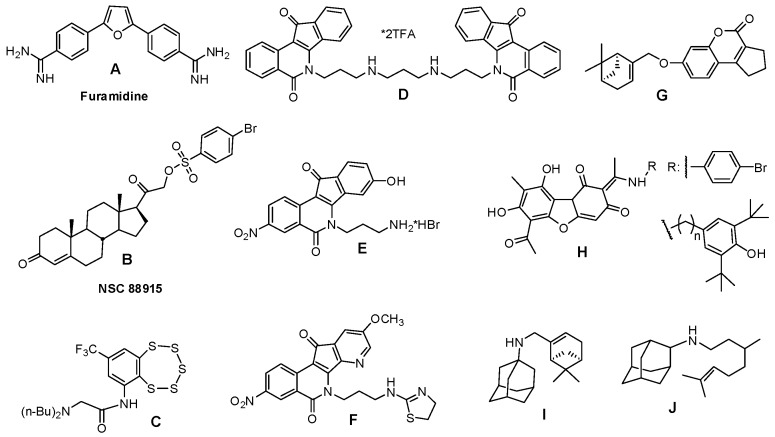
Structures of reported Tdp1 inhibitors.

Several studies reported the development of specific Tdp1 inhibitors of different classes, such as diamidines, phosphotyrosine mimetics, indenoisoquinolines and benzopentathiepines as well as semisynthetic compounds based on natural products (Figure 1) [3,5,10,11,22,23,24,25,26,27,28,29,30]. Commercially available furamidine (**A**) (Figure 1), a bisbenzamidine derivative belonging to the diamidines family, effectively inhibits Tdp1 at low micromolar concentrations both with single- and double-stranded substrates of DNA [23]. Compound **NSC 88915** (**B**) (Figure 1), a conjugate of progesterone and 4-bromobenzenesulfonic acid with relatively high inhibitory characteristics for Tdp1 (IC_50_ value is 7.7 μM), is a representative of phosphotyrosine mimetics [24]. The benzopentathiepines have IC_50_ values in the range of 0.2–6.0 μM. Compound (**C**) containing the dibuthylamino group is the most active (IC_50_ 0.22 μM) [25]. Indenoisoquinoline derivatives were discovered as dual Top1-Tdp1 inhibitors (**D**, **E**) [11,26] and triple Top1–Tdp1–Tdp2 inhibitors (**F**) [27]. The structures of Tdp1 inhibitors based on natural products are represented by coumarin derivatives (**G**) [28], usnic acid derivatives (**H**) [29], and aminoadamantanes containing monoterpene-derived fragments (**I**) and (**J**) [30].

This work was aimed at creating and developing a new class of potent inhibitors against Tdp1 and identifying structure-activity relationships based on the series of bile acids’ derivatives. The proposed inhibitors of Tdp1 belong to the new chemical class, expanding the arsenal of structurally diverse inhibitors, thereby increasing the likelihood of successful progress with at least a part of them.

## 2. Results and Discussion

### 2.1. Virtual Screening

A virtual screen was conducted to identify novel ligands against Tdp1 protein. The crystal structure (1MU7, 2.0 Å) [31,32] was used for docking against known Tdp1 inhibitors, shown in Figure 1, to observe predicted hydrogen bonding interactions and poses as there is no crystal structure with a co-crystallized ligand available. 

**Scheme 1:**
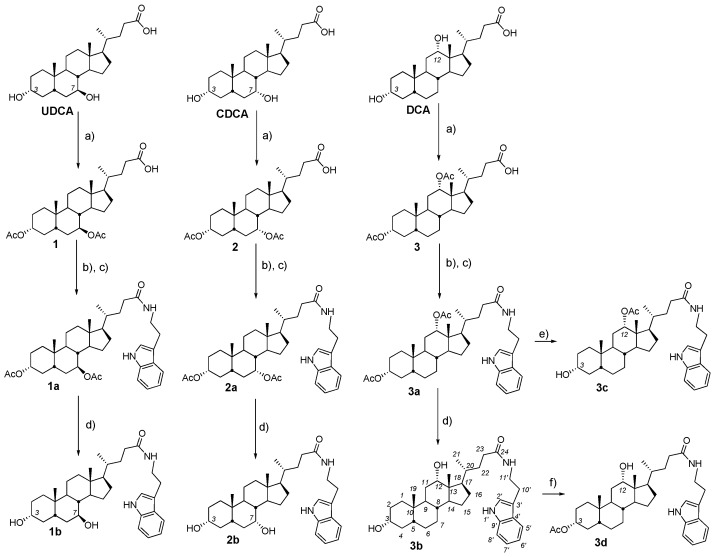
Synthesis of compound **1a**,**b, 2a**,**b** and **3a**,**b** that combine the residues of bile acids and tryptamine: reagent and conditions: (a) Ac_2_O, DMAP, CH_2_Cl_2_ (RT); (b) (COCl)_2_, DMF, CH_2_Cl_2_ (0 °C); (c) tryptamine, NEt_3_, CH_2_Cl_2_ (0 °C, RT); (d) KOH, MeOH (reflux); (e) KOH, MeOH (RT); (f) Ac_2_O, AcOH.

From the natural product library of «InterBioScreen», 9 × 10^3^ compounds were selected for screening. The GoldScore (GS), ChemScore (CS), Chem Piecewise Linear Potential (ChemPLP), and Astex Statistical Potential (ASP) scoring functions were used to assess the binding of the ligands. The scores predicted by these functions reflect the binding energy between the ligands and the protein. In general, the higher the number produced by the scoring functions, the greater chance enhanced efficacy is predicted for the ligand. The virtual screen was done in two phases using a relatively low screening efficiency (30%) intended to weed out compounds that were unlikely to fit the binding pocket of Tdp1, followed by a more robust (100%) search for the remaining ligands (see Methodology for further detail). First, all ligands were screened and those with no or weak predicted hydrogen bonding (<1) were eliminated as well as those with low predicted binding energies (GS < 46, CS < 23, ChemPLP < 55 and ASP < 33). This left 998 candidates, which were screened again with a high search efficiency and again were eliminated based on their binding scores (GS < 50, CS < 25, ChemPLP < 61 and ASP < 34) and poor hydrogen bonding (<1), resulting in 143 candidates. The remaining compounds were visually inspected and selected based on the following criteria: at least three of the four scoring functions agree on a pose; the ability to fill at least one of the two hydrophobic domains, which made up the binding site and had a predicted hydrogen bonding interaction. The results from the visual inspection showed most compounds had a predicted hydrogen bonding interaction with either histidines (263 and 493) or lysines (265 and 495), which were indicated by the literature to be key interactions for successful binding [5]. Other hydrogen bonding was also seen with Asp 283, 288 and 516, Ser 399, 400, 459 and 518, Tyr 204. Based on the listed criteria, sixteen compounds were selected for further testing. Of those compounds, **1b**, shown in Scheme 1, was found to be active against Tdp1 at the low concentration of 0.3 μM.

### 2.2. Synthesis and Testing of Bile Acids Tryptamides

Bile acids (BAs), steroidal molecules synthesized from cholesterol, are widespread in nature and possess a high enantiomeric purity and a broad spectrum of native biological activities (anti-inflammatory, antiviral, anticancer, immunostimulatory) [33,34,35,36]. All of these make BAs interesting and are perspective starting materials for organic synthesis in order to obtain new derivatives with new biological properties [37,38,39].

The commercially available bile acids containing two hydroxy-groups in a steroid framework (ursodeoxycholic acid (3α,7β-dihydroxy-5β-cholan-24-oic acid) **UDCA**; chenodeoxycholic acid (3α,7α-dihydroxy-5β-cholan-24-oic acid) **CDCA,** epimer of **UDCA** at C-7, and deoxycholic acid (3α,12α-dihydroxy-5β-cholan-24-oic acid) **DCA**) were chosen as starting compounds (Scheme 1). At the first step, we resynthesized compound **1b**, and also obtained tryptamides of other bile acids—chenodeoxycholic acid (compound **2b**), and deoxycholic acid (compound **3b**) (Scheme 1). The bile acids were transformed into the target compounds (**1b**, **2b** and **3b**) by a four-step synthesis that included the following stages: (1) acylation of hydroxyl groups in the steroid framework; (2–3) amidation of bile acid chloride by intermediate interaction with tryptamine; (4) the deprotection of hydroxyl groups by treatment with KOH in methanol (Scheme 1).

For testing, we used recently designed oligonucleotide biosensors for the real-time detection of Tdp1 activity based on the ability of Tdp1 to remove fluorophore quenchers from the 3′-end of DNA [27]. The hexadecameric oligonucleotide carried 5(6)-carboxyfluorescein (FAM) at the 5′-end and fluorophore quencher BHQ1 (Black Hole Quencher-1) at the 3′-end. Tdp1 inhibitors prevent the removal of fluorophore quenchers, thus reducing fluorescence intensity.

All the target compounds, **1b**, **2b** and **3b,** were found to exhibit similar activity (IC_50_~2.6 μM), while the activity of the commercial sample of compound **1b** was significantly better (IC_50_ = 0.3 μM) (Table 1).

Analyzing the scheme of the tryptamides synthesis, we proposed that the commercial sample may contain impurities with protected hydroxyl groups. This is why we also tested intermediates **1a**, **2a** and **3a** containing acetoxy groups. And indeed, the derivatives **1a**, **2a,** and **3a** with two acetoxy groups were able to inhibit Tdp1 within a concentration range IC_50_ = 0.32–0.65 μM. Since we did not observe a significant difference in the activity of compounds **1a**–**3a**, **1b**–**3b** and **DCA** is the cheapest bile acid among those that we used in this work, we focused our further studies on **DCA** transformations.

Further, in order to study the influence of functional groups and their location in the steroid framework, we synthesized the tryptamide derivatives of **DCA 3c** and **3d** containing only one acetoxy group at the 3 or 12 position (Scheme 1). 3-Hydroxy-12-acetoxyderivative **3c** was prepared by reaction of 3,12-diacetoxyderivative, **3a** with KOH in MeOH at room temperature, 3-acetoxy-12-hydroxy derivative **3d** was obtained by acylation of the 3-OH-group of compound **3b**. The selectivity of these reactions is explained by the fact that the hydroxyl groups of the steroid framework possess different reactivity [35,37]. Compounds **3c** and **3d** have also been tested and their inhibitory activity was better than the activity of dihydroxyderivatives **1b**–**3b** but slightly worse than those of diacetoxyderivatives **1a**–**3a** (IC_50_ = 0.95 and 0.48 μM, respectively) (Table 1).

Thus, we can conclude that the replacement of at least one hydroxy-group on the steroid framework by an acetoxy group increased the Tdp1 inhibitory activity, while the location of these functional groups in the steroid framework did not significantly influence the activity of the inhibitors.

### 2.3. Synthesis and Testing of Deoxycholic Acid Amides

**Scheme 2:**
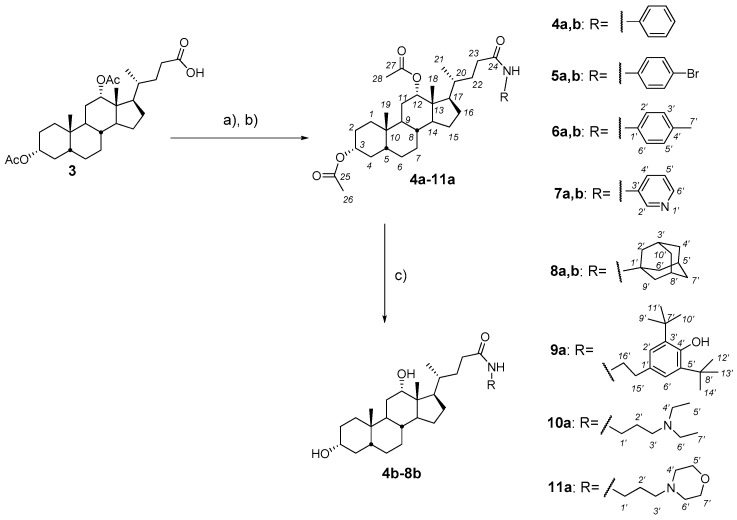
Synthesis of deoxycholic acid amides **4a**,**b**–**8a**,**b** and **9a**–**11a**: (**a**) (COCl)_2_, DMF, CH_2_Cl_2_ (0 °C); (**b**) tryptamine, NEt_3_, CH_2_Cl_2_ (0 °C, RT); (**c**) KOH, MeOH (reflux).

To study the effect of the amide moiety of conjugates on the inhibitory activity, we synthesized the set of amides by reacting **DCA** with various amines. Compounds **4a**,**b**, **5a**,**b**, **6a**,**b**, **7a**,**b**, and **8a**,**b** were obtained with aromatic amines (aniline, *p*-bromoaniline, *p*-methylaniline and 3-aminopyridine) (Scheme 2). Compound **9a** was synthesized using aromatic amine 4-(2-aminoethyl)-2,6-bis-*t*-butylphenol containing C2-linker. Compounds **8a**,**b**, **10a,** and **11a** were synthesized by the reaction with aliphatic amines (1-aminoadamantane, *N,N*-dimethylethanediamine and 2-morpholinethanamine). 

The results from the Tdp1 assay for amides of **DCA** using the FAM-BHQ1 biosensor are shown in Table 2. Compounds containing acetoxy groups in the steroid framework (index **a**) were 3–10 fold more active than the corresponding derivatives containing hydroxyl groups (index **b**). The derivatives with either hydrophobic (adamantane derivative **8a**, substituted anilines **4a**–**6a**) or weak acidic (indole **3a** or phenol **9a** moieties) properties were more active than the derivatives with basic properties (aliphatic amines **10a**, **11a**, as well as pyridine **8a**). 

A study of the cytotoxic activity of diacetoxyderivatives on the human mammary adenocarcinoma (MCF-7) and human colon carcinoma (HCT-116) cells was carried out using the MTT-test. It showed that the toxicity of the target compounds was absent or was insignificant throughout the range of studied concentrations (up to 100 μM). 

### 2.4. Molecular Modeling

The twenty-one derivatives of the bile acid were docked against the binding pocket of the Tdp1 crystal structure (PDB ID: 1MU7, resolution 2.0 Å) [32] with good predicted affinity by the scoring functions used (see Table S1 in the Supplementary Materials). Highly active inhibitors like **3a** tend to have higher scores than less active ligands, as predicted by ChemPLP and GS.

**Figure 2:**
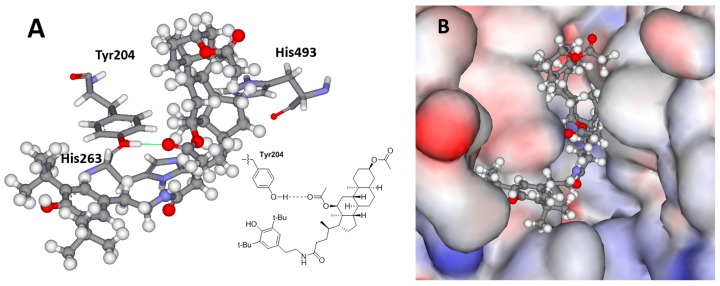
The docked configuration of **9a** to the binding site of Tdp1 using the ChemPLP scoring function. (**A**) Hydrogen bond is depicted as a green line between the ligand and the amino acid TYR204. (**B**) The protein surface is rendered. The steroid group is inserted in a lipophilic pocket and the acyl groups are exposed to the water environment. Red depicts a positive partial charge on the surface, blue depicts a negative partial charge and grey shows the neutral/lipophilic areas.

Many studies suggest that blocking the histidine amino acid residues 263 and 493 is important [28,32]. As can be seen in Figure 2A for the predicted pose of **9a**, it denies access to both of these histidine residues, reducing the enzymatic activity of Tdp1. Only one hydrogen bond is predicted between **9a** and the protein, i.e., Tyr204 and the carbonyl group of the acetate moiety attached to carbon 12 in the steroid scaffold. A good fit into the binding pocket is observed (see Figure 2B) with the steroid scaffold occupying the cleft containing His263 and 493. The acetate groups are both directed towards the aqueous phase and the di-tert-butyl substituted phenol is partially placed in a shallow lipophilic pocket. In general, a plausible binding mode is generated, which can explain the excellent efficacy of **9a**. 

There is a clear trend for enhanced activity with the acetoxy groups as compared to the hydroxyl moiety when derivatives **1a**–**8a** and **1b**–**8b** are studied (see Table 1 and Table 2). The modelling reveals that the acetoxy group at C3 is pointing into the aqueous phase with both the oxygen atoms able to form hydrogen bonds with the water molecules, whereas the methyl moiety is adjacent to a lipophilic surface, as shown in Figure 2B. The hydroxyl group, however, can also form hydrogen bonds with water but no lipophilic contacts. As for the C12 acetoxy substituent, it forms a hydrogen bond with Tyr204 for the **1a**–**8a** series, which the hydroxyl does not do for the **1b**–**8b** derivatives. Thus, the lipophilic contact and hydrogen bond via the acetoxy substituents appear to be important for enhanced binding of the bile acid derivatives to Tdp1.

The molecular descriptors are given in Table S2 in the Supplementary Materials. Unsurprisingly, the MW is high with most of the ligand having >500 g mol^−1^, which is outside drug-like chemical space. However, none of the ligands are outside the 800 g mol^−1^ limit, they are within Known Drug Space (KDI [40], see Table S3 in the Supplementary Materials). All of the bile acids are outside drug-like chemical space, except **7a**, **7b**, **10a** and **11a** in terms of log P. The most active compound **9a** has a predicted log P of 9.0, which is even outside KDI and can explain the high efficacy of this ligand. The large size and relatively high lipophilicity is *not* surprising since the bile acids have a steroid core, which is large and greasy. The hydrogen bond donors and acceptors are all within drug-like chemical space, for the rotatable bonds in a number of ligands (**1a**, **2a**, **3b**, **9a**, **10a** and **11a**) are outside this boundary but within the KDI. 

## 3. Materials and Methods 

### 3.1. Chemicals and Reagents

Elemental analyses were determined on an Automatic CHNS-analyser EURO EA3000. Analyses indicated by the symbols of the elements were within ± 0.4% of the theoretical values. Melting points were determined on a METTLER TOLEDO FP900 thermosystem and are uncorrected. The element composition of the products was determined from high-resolution mass spectra recorded on a DFS (double focusing sector) Thermo Electron Corporation instrument. Optical rotations were measured with a PolAAr 3005 polarimeter. ^1^H and ^13^C-NMR spectra were measured on Bruker spectrometers: AV-600 (operating frequency 600.30 MHz for ^1^H and 150.95 MHz for ^13^C) and DRX-500 (500.13 MHz for ^1^H and 125.76 MHz for ^13^C) using CDCl_3_ solutions of the substances. The chemical shifts were recorded in *δ* (ppm) using the *δ* 7.24 of CHCl_3_ (^1^H-NMR) and *δ* 76.90 (^13^C-NMR) as internal standards. Chemical shift measurements are given in ppm and the coupling constants (*J*) in hertz (Hz). The structure of the compounds was determined by NMR using the standard one-dimensional and two-dimensional procedures (^1^H-^1^H COSY, ^1^H-^13^C HMBC/HSQC, ^13^C-^1^H HETCOR/COLOC). The purity of the final compounds and intermediates for biological testing was confirmed to be more than 95%, as determined by the HPLC analysis. HPLC analyses were carried out on a MilichromA-02, using a ProntoSIL 120-5-C18 AQ column (BISCHOFF, 2.0 × 75 mm column, grain size 5.0 lm). Mobile phase: Millipore purified water with 0.1% trifluoroacetic acid with a linear gradient of 0–100% methanol at a flow rate of 150 µL/min at 35 °C and UV detection at 210, 220, 240, 260, and 280 nm. Flash column chromatography was performed with silica gel (Merck, 60–200 mesh). All reactions’ courses were monitored by TLC analysis using Merck 60 F_254_ silica gel on aluminum sheets, eluent CHCl_3_-MeOH (20:3) or CHCl_3_-AcOEt (20:3). 

Ursodeoxycholic acid (99%), tryptamine (98%), 3-aminopyridine (99%), 1-aminoadamantane hydrochloride (99%) were purchased from ACROS Organics, chenodeoxycholic acid (>98%) was purchased from ROTH, deoxycholic acid 99%, oxalyl chloride (98%) were purchased from abcr GmbH & Co. KG, *N*^1^,*N*^1^-diethylaminopropan-1,3-diamine (99+%) and 3-morpholinopropan-1-amine (98%) were purchased from Aldrich. All solvents used in the reactions were previously purified and dried according to the previously reported procedures. 

Diacetoxy derivatives of bile acids 1–3 were synthesized according to the known literature method [41] by treatment of a bile acid, such as ursodeoxycholic (UDCA), chenodeoxycholic (CDCA), and deoxycholic (DCA) with acetic anhydride in the presence of 4,4-dimethylaminopyridine (DMAP) in anhydrous dichloromethane with yield 95%.

(a) *General procedures for compound* **1a**–**11a**
  (1) Oxalyl chloride (6.0 equiv.) and a few drops DMF were added at 0 °C to a solution of diacetoxy bile acid (1–3) (1.0 equiv.), correspondently, in dry CH_2_Cl_2_. The reaction mixture was stirred for a further 3 h at 0–5 °C, diluted with benzene and concentrated in vacuum. Then CH_2_Cl_2_.was added to the reaction mixture to give a solution of bile acid chloride.
  (2) The resulting solution bile acid chloride (1.0 equiv.) was added dropwise at 0 °C to a solution of appropriate amine (1.2 equiv.) and NEt_3_ (1.5 equiv.) in dry CH_2_Cl_2_. The reaction mixture was stirred for a further 18 h at room temperature, diluted with AcOEt (20 mL) and H_2_O was added. The organic layer was separated and the aqueous layer was extracted with AcOEt (2 × 30 mL). The combined organic layers were washed with brine and dried over calcined MgSO_4_. The solvent was removed to give an amorphous solid.
(b) *General procedures for compound* **1b**–**8b**
  A solution of the appropriate amide derivatives (**1a**–**8a**) (1 equiv.)—containing acetoxy groups in the steroid framework—in MeOH was treated with KOH (6 equiv.), refluxed for 2–6 h (monitored by TLC), concentrated under reduced pressure, diluted with H_2_O (up to ~50–60 mL), acidified with 5% HCl solution to pH 3–4, and extracted with mixture CH_2_Cl_2_Et_2_O (1:3 *v*/*v*) (3 × 30 mL). The combined organic layers were washed with a saturated solution of NaHCO_3_, brine and dried over calcined MgSO_4_. The solvent was removed to give an amorphous solid.

#### 3.1.1. *N*-(2′-(1H-Indol-2-yl)-ethyl)-3α,7β-diacetoxy-5β-cholan-24-amide (**1a**)

Crude product as an amorphous white solid (1.52 g, 97%) was obtained from acid chloride intermediate (preparing by reaction diacetoxy derivative 1 (1.20 g, 2.5 mmol) and oxalyl chloride (1.3 mL, 15.0 mmol) in CH_2_Cl_2_ (10 mL)) and tryptamine (0.48 g, 3.0 mmol) with addition of NEt_3_ (0.6 mL, 3.8 mmol) in CH_2_Cl_2_ (10 mL) according to the general procedure. The crude product was purified by flash column chromatography (silica gel, 0–5% MeOH in CHCl_3_) to yield compound **1a** (1.25 g, 80%) as an amorphous white. M.p. 107.6 °C [decomposition]. [$\alpha_{D}^{23}$] + 42° (*c* 0.20 g/100 mL; CHCl_3_). HRMS: *m*/*z* calcd for C_38_H_54_N_2_O_5_: 618.4027; found: 618.4020. Anal. Calcd. For C_38_H_54_N_2_O_5_: C, 73.75; H, 8.80; N, 4.53; O, 12.93; found C, 73.68; H, 8.76; N, 4.31. ^1^H-NMR (CDCl_3_, 500 MHz): δ = 8.36 (br s, 1H, NH-1′), 7.57 (d, 1H, *J*_5′,6′_ = 7.7, H-5′), 7.35 (d, 1H, *J*_8′,7′_ = 8.0, H-8′), 7.18 (dd, 1H, *J*_7′,6′_ = 7.4, H-7′), 7.09 (dd, 1H, *J*_6′,5′_ = 7.4, H-6′), 7.00 (br s, 1H, H-2′), 5.65 (m, 1H, C(O)NH), 4.73 (s, 1H, H-7(α)), 4.63 (m, 1H, H-3(β)), 3.57 (m, 2H, CH_2_-11′), 2.95 (m, 2H, CH_2_-10′), 2.00 (s, 3H, CH_3_-26), 1.96 (s, 3H, CH_3_-28), 0.93 (s, 3H, CH_3_-18), 0.85 (d, 3H, *J*_21,20_ = 6.2, CH_3_-21), 0.62 (s, 3H, CH_3_-19). ^13^C-NMR (CDCl_3_, 125 MHz): δ = 173.46 (s, C-24), 170.52 (s, C-25), 170.44 (s, C-27), 136.25 (s, C-9′), 127.15 (s, C-4′), 121.93 (d, C-2′), 121.93 (d, C-7′), 119.12 (d, C-5′), 118.44 (d, C-6′), 112.60 (s, C-3′), 111.16 (d, C-8′), 73.40 (d, C-7), 73.40 (d, C-3), 54.95, 54.75, 43.32, 41.79, 39.70, 39.66, 39.61, 39.12, 35.10, 34.26, 33.74, 33.39, 32.65, 32.65, 31.54, 28.22, 26.15, 25.38, 25.15 (t, C-10′), 23.00, 21.66, 21.21, 20.96, 18.25(q, C-21), 11.86(q, C-18).

#### 3.1.2. *N*-(2′-(1H-Indol-2-yl)-ethyl)-3α,7β-dihydroxy-5β-cholan-24-amide (**1b**)

Crude product as an amorphous white solid (0.55 g, 92%) was prepared from diacetoxy derivative **1a** (0.69 g, 1.1 mmol) and KOH (0.37 g, 6.6 mmol) in MeOH (25 mL) according to the general procedure. An analytically pure sample of **1b** (0.22 g, 37%) was obtained by flash column chromatography (silica gel, 0–5% MeOH in CHCl_3_). M.p. 114.9 °C [decomposition]. [$\alpha_{D}^{23}$] + 28° (*c* 0.20 g/100 mL; CHCl_3_). HRMS: *m*/*z* calcd for C_34_H_50_N_2_O_3_: 534.3816; found: 534.3813. Anal. Calcd. for C_34_H_50_N_2_O_3_: C, 76.36; H, 9.42; N, 5.24; O, 8.98; found C, 76.43; H, 9.64; N, 4.89. ^1^H-NMR (CDCl_3_, 500 MHz): δ = 8.96 (br s, 1H, NH-1′), 7.53 (d, 1H, *J*_5′,6′_ = 7.5, H-5′), 7.33 (d, 1H, *J*_8′,7′_ = 8.1, H-8′), 7.13 (m, 1H, H-7′), 7.05 (m, 1H, H-6′), 6.97 (br s, 1H, H-2′), 5.97 (m, 1H, C(O)NH), 3.49 (m, 4H: H-3(β), H-7(α), CH_2_-11′), 2.91 (m, 2H, CH_2_-10′), 0.87 (s, 3H, CH_3_-18), 0.82 (d, 3H, *J*_21,20_ = 6.0, CH_3_-21), 0.58 (s, 3H, CH_3_-19). ^13^C-NMR (CDCl_3_, CD_3_OD, 125 MHz): δ = 174.00 (s, C-24), 136.17 (s, C-9′), 127.06 (s, C-4′), 121.98 (d, C-2′), 121.73 (d, C-7′), 119.00 (d, C-5′), 118.35 (d, C-6′), 112.25 (s, C-3′), 111.17 (d, C-8′), 71.00 (d, C-3), 70.95 (d, C-7), 55.49, 54.60, 43.46, 43.33, 42.20, 39.88, 39.49, 39.00, 36.85, 36.65, 35.17, 34.69, 33.82, 33.32, 31.68, 29.85, 28.40, 25.01, 23.17 (q, C-19), 20.94, 18.21 (q, C-21), 11.87 (q, C-18).

#### 3.1.3. *N*-(2′-(1H-Indol-2-yl)-ethyl)-3α,7a-diacetoxy-5β-cholan-24-amide (**2a**)

Crude product as an amorphous white solid (1.40 g, 89%) was obtained from acid chloride intermediate (preparing by reaction diacetoxy derivative 2 (1.20 g, 2.5 mmol) and oxalyl chloride (1.3 mL, 15.0 mmol) in CH_2_Cl_2_ (10 mL)) and tryptamine (0.48 g, 3.0 mmol) with addition of NEt_3_ (0.6 mL, 3.8 mmol) in CH_2_Cl_2_ (10 mL) according to the general procedure. The crude product was purified by flash column chromatography (silica gel, 0–1% MeOH in CHCl_3_) to yield compound **2a** (1.10 g, 70%) as an amorphous white. M.p. 98.7 °C [decomposition]. [$\alpha_{D}^{23}$] + 8° (*c* 0.20 g/100 mL; CHCl_3_). HRMS: *m*/*z* calcd for C_38_H_54_N_2_O_5_: 618.4027; found: 618.4023. Anal. Calcd. For C_38_H_54_N_2_O_5_: C, 73.75; H, 8.80; N, 4.53; O, 12.93; found C, 73.54; H, 8.63; N, 4.55. ^1^H-NMR (CDCl_3_, 500 MHz): δ = 8.47 (br s, 1H, NH-1′), 7.56 (d, 1H, *J*_5′,6′_ = 7.8, H-5′), 7.34 (d, 1H, *J*_8′,7′_ = 8.1, H-8′), 7.17 (dd, 1H, *J*_7′,6′_ = 7.6, H-7′), 7.09 (dd, 1H, *J*_6′,5′_ = 7.4, H-6′), 6.99 (s, 1H, H-2′), 5.67 (s, 1H, C(O)NH), 4.84 (s, 1H, H-7(β)), 4.55 (m, 1H, H-3(β)), 3.56 (d, 2H, *J* = 5.8, CH_2_-11′), 2.94 (m, 2H, CH_2_-10′), 2.02 (s, 3H, CH_3_-26), 2.01 (s, 3H, CH_3_-28), 0.89 (s, 3H, CH_3_-18), 0.86 (d, 3H, *J*_21,20_ = 6.0, CH_3_-21), 0.59 (s, 3H, CH_3_-19). ^13^C-NMR (CDCl_3_, 125 MHz): δ = 173.49 (s, C-24), 170.54 (s, C-25), 170.37 (s, C-27), 136.25 (s, C-9′), 127.16 (s, C-4′), 121.94 (d, C-2′), 121.89 (d, C-7′), 119.19 (d, C-5′), 118.47 (d, C-6′), 112.66 (s, C-3′), 111.16 (d, C-8′), 74.02 (d, C-3), 71.09 (d, C-7), 55.54 (d, C-17), 50.16 (d, C-14), 42.47 (s, C-13), 40.72 (d, C-5), 39.63, 39.26, 37.66 (d, C-8), 35.22, 34.68, 34.58, 34.41, 33.83, 33.40, 31.56, 31.12, 27.86, 26.58, 25.13 (t, C-10′), 23.35, 22.49, 21.46, 21.33, 20.43, 18.17(q, C-21), 11.51 (q, C-18).

#### 3.1.4. *N*-(2′-(1H-Indol-2-yl)-ethyl)-3α,7α-dihydroxy-5β-cholan-24-amide (**2b**).

Crude product as an amorphous white solid (0.55 g, 92%) was prepared from diacetoxy derivative **2a** (0.69 g, 1.1 mmol) and KOH (0.37 g, 6.6 mmol) in MeOH (25 mL) according to the general procedure. An analytically pure sample of **2b** (0.22 g, 37%) was obtained by flash column chromatography (silica gel, 0–5% MeOH in CHCl_3_). M.p. 112.9 °C [decomposition]. [$\alpha_{D}^{23}$] + 5° (*c* 0.20 g/100 mL; CHCl_3_). HRMS: *m*/*z* calcd for C_34_H_50_N_2_O_3_: 534.3816; found: 534.3818. Anal. Calcd. for C_34_H_50_N_2_O_3_: C, 76.36; H, 9.42; N, 5.24; O, 8.98; found C, 75.94; H, 9.06; N, 4.84. ^1^H-NMR (CDCl_3_, 500 MHz): δ = 8.73 (br s, 1H, NH-1′), 7.55 (d, 1H, *J*_5′,6′_ = 7.8, H-5′), 7.35 (d, 1H, *J*_8′,7′_ = 7.8, H-8′), 7.16 (dd, 1H, *J*_7′,6′_ = 7.0, *J*_7′,8′_ = 7.8, H-7′), 7.08 (dd, 1H, *J*_6′,5′_ = 7.8, H-6′), 7.00 (br s, 1H, H-2′), 5.80 (br s, 1H, C(O)NH), 3.80 (br s, 1H, H-7(β)), 3.56 (m, 2H, CH_2_-11′), 3.43 (m, 1H, H-3(β)), 2.93 (m, 2H, CH_2_-10′), 0.86 (s, 3H, CH_3_-18), 0.59 (d, 3H, CH_3_-21), 0.59 (s, 3H, CH_3_-19). ^13^C-NMR (CDCl_3_, 125 MHz): δ = 173.82 (s, C-24), 136.32 (s, C-9′), 127.17 (s, C-4′), 122.12 (d, C-2′), 121.85 (d, C-7′), 119.15 (d, C-5′), 118.48 (d, C-6′), 112.55 (s, C-3′), 111.27 (d, C-8′), 71.79 (d, C-3), 68.33 (d, C-7), 55.50 (d, C-17), 50.20 (d, C-14), 42.44 (s, C-13), 41.29, 39.56, 39.49, 39.43, 39.18, 35.27, 35.15, 34.85, 33.31, 32.65, 31.65, 30.86, 30.39, 28.03, 25.14, 23.49, 22.64 (q, C-19), 20.41, 18.22 q, C-21),, 11.60 (q, C-18).

#### 3.1.5. *N*-(2′-(1H-Indol-2-yl)-ethyl)-3α,12a-diacetoxy-5β-cholan-24-amide (**3a**)

Crude product as an amorphous white solid (2.1 g, 82%) was obtained from acid chloride intermediate (preparing by reaction diacetoxy derivative 3 (2.0 g, 4.1 mmol) and oxalyl chloride (2.0 mL, 24.6 mmol) in CH_2_Cl_2_ (10 mL)) and tryptamine (0.79 g, 4.9 mmol) with addition of NEt_3_ (0.9 mL, 6.2 mmol) in CH_2_Cl_2_ (10 mL) according to the general procedure. The crude product was purified by flash column chromatography (silica gel, 0–1% MeOH in CHCl_3_) to yield compound **3a** (0.94 g, 45%) as an amorphous white. M.p 99.2 °C [decomposition]. [$\alpha_{D}^{23}$] + 72° (*c* 0.20 g/100 mL; CHCl_3_). HRMS: *m*/*z* calcd for C_38_H_54_N_2_O_5_: 618.4027; found: 618.4021. Anal. Calcd. for C_38_H_54_N_2_O_5_: C, 73.75; H, 8.80; N, 4.53; O, 12.93; found C, 73.37; H, 8.68; N, 4.18. ^1^H-NMR (CDCl_3_, 300 MHz): δ = 8.63 (br s, 1H, NH-1′), 7.55 (d, 1H, *J*_5′,6′_ = 7.8, H-5′), 7.33 (d, 1H, *J*_8′,7′_ = 8.0, H-8′), 7.15 (dd, 1H, *J*_7′,6′_ = 7.2, H-7′), 7.06 (dd, 1H, *J*_6′,5′_ = 7.4, H-6′), 6.97 (s, 1H, H-2′), 5.60 (m, 1H, C(O)NH), 5.05 (s, 1H, H-12(β)), 4.67 (m, 1H, H-3(β)), 3.54 (m, 2H, CH_2_-11′), 2.92 (m, 2H, CH_2_-10′), 2.06 (s, 3H, CH_3_-26), 2.01 (s, 3H, CH_3_-28), 0.88 (s, 3H, CH_3_-18), 0.71 (d, 3H, *J*_21,20_ = 6.4, CH_3_-21), 0.67 (s, 3H, CH_3_-19). ^13^C-NMR (CDCl_3_, 125 MHz): δ = 173.30 (s, C-24), 170.44 (s, C-25), 170.37 (s, C-27), 136.32 (s, C-9′), 127.25 (s, C-4′), 122.03 (d, C-2′), 121.90 (d, C-7′), 119.32 (d, C-5′), 118.55 (d, C-6′), 112.86 (s, C-3′), 111.16 (d, C-8′), 75.80 (d, C-12), 74.08 (d, C-3), 49.28, 47.64, 44.89, 41.70, 39.67 (t, C-11′), 35.54, 34.67, 34.59, 34.28, 33.90, 33.55, 32.14, 31.47, 27.18, 26.75, 26.50, 25.73, 25.52, 25.19 (t, C-10′), 23.28, 22.92 (q, CH_3_-C(O)O), 21.32 (q, C-19), 21.25 (q, CH_3_-C(O)O), 17.49 (q, C-21), 12.29 (q, C-18).

#### 3.1.6. *N*-(2′-(1H-Indol-2-yl)-ethyl)-3α,12α-dihydroxy-5β-cholan-24-amide (**3b**)

Crude product as an amorphous white solid (0.35 g, 77%) was prepared from diacetoxy derivative **3a** (0.52 g, 0.9 mmol) and KOH (0.3 g, 5.4 mmol) in MeOH (8 mL) according to the general procedure. An analytically pure sample of **3b** (0.20 g, 44%) was obtained by flash column chromatography (silica gel, 0–8% MeOH in CHCl_3_). M.p. 117.7 °C [decomposition]. [$\alpha_{D}^{27}$] + 34° (*c* 0.20 g/100 mL; CH_3_OH). HRMS: *m*/*z* calcd for C_34_H_50_N_2_O_3_: 534.3816; found: 534.3815. ^1^H-NMR (CDCl_3_, 300 MHz): δ = 8.63 (br s, 1H, NH-1′), 7.56 (d, 1H, *J*_5′,6′_ = 7.8, H-5′), 7.36 (d, 1H, *J*_8′,7′_ = 8.2, H-8′), 7.17 (m, 1H, H-7′), 7.08 (m 1H, H-6′), 7.00 (br s, 1H, H-2′), 5.81 (br s, 1H, C(O)NH), 3.90 (s, 1H, H-12(β)), 3.55 (m, 3H: H-3(β), CH_2_-11′), 2.93 (m, 2H, CH_2_-10′), 0.89 (d, 3H, *J*_21,20_ = 6.0, CH_3_-21), 0.87 (s, 3H, CH_3_-18), 0.61 (s, 3H, CH_3_-19). ^13^C-NMR (CDCl_3_, 75 MHz): δ = 173.70 (s, C-24), 136.32 (s, C-9′), 127.23 (s, C-4′), 122.11 (d, C-2′), 121.93 (d, C-7′), 119.23 (d, C-5′), 118.54 (d, C-6′), 112.74 (s, C-3′), 111.27 (d, C-8′), 73.03 (d, C-12), 71.59 (d, C-3), 48.10, 46.91, 46.32, 41.91, 39.60 (t, C-11′), 36.21, 35.83, 35.08, 33.96, 33.50, 33.33, 31.54, 30.26, 28.47, 27.34, 26.98, 25.98, 25.17 (t, C-10′), 23.51, 22.99 (q, C-19), 17.27 (q, C-21), 12.61 (q, C-18).

#### 3.1.7. *N*-(2′-(1H-Indol-2-yl)-ethyl)-3α-hydroxy-12α-acetoxy-5β-cholan-24-amide (**3c**)

A mixture of **3a** (0.65 g, 1.0 mmol) and KOH (0.17 g, 3.0 mmol) in methanol (5 mL) was stirred at room temperature (monitored by TLC), concentrated under reduced pressure, diluted with H_2_O (up to ~15 mL), acidified with 5% HCl solution to pH 3–4, and extracted with mixture CH_2_Cl_2_Et_2_O (1:3 *v*/*v*) (3 × 20 mL). The combined organic layers were washed with saturated solution of NaHCO_3_, brine and dried over calcined MgSO_4_. The solvent was removed to give an amorphous solid **3c** (0.50 g, 85%). An analytically pure sample of 3c (0.45 g, 76%) was obtained by flash column chromatography (silica gel, 30–55% AcOEt in hexane). M.p. 106.5 °C [decomposition]. HRMS: *m*/*z* calcd for C_36_H_52_N_2_O_4_: 576.3922; found: 576.3927. ^1^H-NMR (CDCl_3_, 300 MHz): δ = 8.30 (br s, 1H, NH-1′), 7.57 (d, 1H, *J*_5′,6′_ = 7.8, H-5′), 7.36 (d, 1H, *J*_8′,7′_ = 8.0, H-8′), 7.18 (m, 1H, H-7′), 7.09 (m 1H, H-6′), 7.09 (br s, 1H, H-2′), 5.67 (br s, 1H, C(O)NH), 5.03 (s, 1H, H-12(β)), 3.57 (m, 3H: H-3(β), CH_2_-11′), 2.95 (t, 2H, CH_2_-10′, *J*_10′,11′_*=* 6.4), 2.03 (s, 3H, CH_3_-28), 0.87 (s, 3H, CH_3_-18), 0.74 (d, 3H, *J*_21,20_ = 6.0, CH_3_-21), 0.66 (s, 3H, CH_3_-19). ^13^C-NMR (CDCl_3_, 75 MHz): δ = 173.42 (s, C-24), 170.54 (s, C-27), 136.29 (s, C-9′), 127.21 (s, C-4′), 122.03 (d, C-2′), 121.92 (d, C-7′), 119.32 (d, C-5′), 118.54 (d, C-6′), 112.80 (s, C-3′), 111.17 (d, C-8′), 75.84 (d, C-12), 71.56 (d, C-3), 49.25, 47.56, 44.85, 41.83, 39.66 (t, C-11′), 36.10, 35.56, 34.84, 34.63, 34.28, 33.88, 33.88, 31.45, 30.29, 27.17, 26.91, 25.81, 25.49, 25.16 (t, C-10′), 23.30, 22.96 (q, C-19), 21.32 (q, C-28), 17.47 (q, C-21), 12.29 (q, C-18).

#### 3.1.8. *N*-(2′-(1H-Indol-2-yl)-ethyl)-3α-acetoxy-12α-hydroxy-5β-cholan-24-amide (**3d**)

To a solution of **3a** (0.14 g, 0.22 mmol) in dry CH_2_Cl_2_ (10 mL) was added Ac_2_O (0.03 mL, 0.33 mmol). The reaction mixture was stirred for a further 3 h at room temperature (monitored by TLC) and added H_2_O (10 mL). The organic layer was separated and the aqueous layer was extracted with AcOEt (2 × 30 mL). The combined organic layers were washed with a saturated solution of NaHCO_3_, brine and dried over calcined MgSO_4_. The solvent was removed to give an amorphous solid **3d** (0.13 g, quantitative yield). An analytically pure sample of 3d (0.09 g, 69%) was obtained by flash column chromatography (silica gel, 30–50% AcOEt in hexane). M.p. 196.7–197.1 °C. Anal. Calcd. for C_36_H_52_N_2_O_4_: C, 74.96; H, 9.09; N, 4.86; O, 11.10; found C, 75.05; H, 8.78; N, 4.65. HRMS: *m*/*z* calcd for C_36_H_52_N_2_O_4_: 576.3922; found: 576.3926. ^1^H-NMR (CDCl_3_, 300 MHz): δ = 8.49 (br s, 1H, NH-1′), 7.56 (d, 1H, *J*_5′,6′_ = 7.8, H-5′), 7.35 (d, 1H, *J*_8′,7′_ = 8.0, H-8′), 7.17 (m, 1H, H-7′), 7.09 (m 1H, H-6′), 6.99 (br s, 1H, H-2′), 5.72 (br s, 1H, C(O)NH), 4.68 (m, 1H, H-3(β)), 3.92 (s, 1H, H-12(β)), 3.56 (m, 2H:, CH_2_-11′, *J*_11′,10′_*=* 6.3), 2.94 (t, 2H, CH_2_-10′, *J*_10′,11′_*=* 6.3), 1.99 (s, 3H, CH_3_-26) 0.85-0.92 [6H: (CH3-21), 0.88 (s, 3H, CH3-18)], 0.62 (s, 3H, CH_3_-19). ^13^C-NMR (CDCl_3_, 75 MHz): δ = 173.70 (s, C-24), 170.59 (s, C-25), 136.28 (s, C-9′), 127.19 (s, C-4′), 121.97 (d, C-2′), 121.93 (d, C-7′), 119.22 (d, C-5′), 118.49 (d, C-6′), 112.69 (s, C-3′), 111.19 (d, C-8′), 74.17 (d, C-3), 72.93 (d, C-12), 48.10, 47.03, 46.28, 41.67, 39.63 (t, C-11′), 35.77, 35.00, 33.94, 33.46, 33.33, 31.99, 31.49, 28.52, 27.28, 26.78, 26.32, 25.84, 25.13 (t, C-10′), 23.41, 22.95 (q, C-19), 21.32 (q, C-26), 17.24 (q, C-21), 12.61 (q, C-18).

#### 3.1.9. *N*-Phenyl-3α,12α-diacetoxy-5β-cholan-24-amide (**4a**)

Crude product as an amorphous white solid (2.0 g, 87%) was obtained from acid chloride intermediate (preparing by reaction diacetoxy derivative 3 (2.0 g, 4.2 mmol) and oxalyl chloride (2.1 mL, 25.2 mmol) in CH_2_Cl_2_ (10 mL)) and aniline (0.45 g, 5.0 mmol) with addition of NEt_3_ (0.9 mL, 6.3 mmol) in CH_2_Cl_2_ (20 mL) according to the general procedure. The crude product was purified by flash column chromatography (silica gel, CHCl_3_) to yield compound **4a** (1.73 g, 75%) as an amorphous white. M.p. 97.5 °C [decomposition]. [$\alpha_{D}^{24.1}$] + 80° (*c* 0.20 g/100 mL; CHCl_3_). HRMS: *m*/*z* calcd for C_34_H_49_NO_5_: 551.3605; found: 551.3604. Anal. Calcd. for C_34_H_49_NO_5_: C, 74.01; H, 8.95; N, 2.54; O, 14.50; Found C, 73.91; H, 8.88; N, 2.78. ^1^H-NMR (CDCl_3_, 300 MHz): δ = 7.48 (d, 2H, *J* = 7.9, H-2′, H-6′), 7.38 (s, 1H, NH), 7.27 (dd, 2H, *J* = *J* = 7.9, H-3′, H-5′), 7.06 (dd, 1H, *J* = 7.4, H-4′), 5.06 (s, 1H, H-12(β)), 4.67 (m, 1H, H-3(β)), 2.38 (m, 1H, H-23), 2.19 (m, 1H, H-23′), 2.08 (s, 3H, CH_3_-26), 2.00 (s, 3H, CH_3_-28), 0.87 (s, 3H, CH_3_-19), 0.81 (d, 3H, *J*_21,20_ = 6.3, CH_3_-21), 0.69 (s, 3H, CH_3_-18). ^13^C MNR (CDCl_3_, 150 MHz): δ = 171.50 (s, C-24), 170.48 (s, C-25), 170.42 (s, C-27), 137.89 (s, C-1′), 128.82 (d, C-3′), 128.82 (d, C-5′), 123.97 (d, C-4′), 119.59 (d, C-2′), 119.59 (d, C-6′), 75.83 (d, C-12), 74.08 (d, C-3), 49.27, 47.71, 44.89, 41.66, 35.51, 34.74, 34.56, 34.24, 33.88, 32.10, 31.33, 27.26, 26.73, 26.48, 25.71, 25.51, 23.29, 22.92 (q, C-19), 21.34 (q, CH_3_-C(O)O), 21.28 (q, CH_3_-C(O)O), 17.52 (q, C-21), 12.33(q, C-18).

#### 3.1.10. *N*-Phenyl-3α,12α-dihydroxy-5β-cholan-24-amide (**4b**)

Crude product as an amorphous white solid (0.43 g, 77%) was prepared from diacetoxy derivative **4a** (0.40 g, 0.7 mmol) and KOH (0.23 g, 4.2 mmol) in MeOH (10 mL) according to the general procedure. An analytically pure sample of **4b** (0.23 g, 68%) was obtained by flash column chromatography (silica gel, 0–2% MeOH in CHCl_3_). M.p. 214.2–216.3 °C. [$\alpha_{D}^{25.6}$] + 35° (*c* 0.20 g/100 mL; CHCl_3_). HRMS: *m*/*z* calcd for C_30_H_45_NO_3_: 467.3394; found: 467.3388. Anal. Calcd. for C_30_H_45_NO_3_: C, 77.04; H, 9.70; N, 2.99; O, 10.26; found C, 77.32; H, 9.39; N, 3.12. ^1^H-NMR (CDCl_3_, 400 MHz): δ = 8.00 (s, 1H, NH), 7.53 (d, 2H, *J* = 7.7, H-2′, H-6′), 7.25 (dd, 2H, *J = J =* 7.8, H-3′, H-5′), 7.04 (dd, 1H, *J* = 7.3, H-4′), 3.97 (s, 1H, H-12(β)), 3.56 (m, 1H, H-3(β)), 2.38 (m, 1H, H-23), 2.22 (m, 1H, H-23′), 0.95 (d, 3H, *J*_21,20_ = 6.0, CH_3_-21), 0.87 (s, 3H, CH_3_-19), 0.64 (s, 3H, CH_3_-18). ^13^C-NMR (CDCl_3_, 100 MHz): δ = 172.38 (s, C-24), 138.21 (s, C-1′), 128.74 (d, C-3′), 128.74 (d, C-5′), 123.75 (d, C-4′), 119.55 (d, C-2′), 119.55 (d, C-6′), 73.10 (d, C-12), 71.67 (d, C-3), 47.66, 46.41, 46.20, 42.00, 36.19, 35.91, 35.21, 35.03, 34.09, 33.62, 33.45, 30.97, 30.67, 28.15, 27.50, 27.19, 26.09, 23.65, 23.02 (q, C-19), 17.34 (q, C-21), 12.48(q, C-18).

#### 3.1.11. *N*-(4′-Bromophenyl)-3α,12α-diacetoxy-5β-cholan-24-amide (**5a**)

Crude product as an amorphous white solid (2.45 g, 92%) was obtained from acid chloride intermediate (preparing by reaction diacetoxy derivative 3 (2.0 g, 4.2 mmol) and oxalyl chloride (2.1 mL, 25.2 mmol) in CH_2_Cl_2_ (10 mL)) and p-bromoaniline (0.86 g, 5.0 mmol) with addition of NEt_3_ (0.9 mL, 6.3 mmol) in CH_2_Cl_2_ (20 mL) according to the general procedure. The crude product was purified by flash column chromatography (silica gel, CHCl_3_) to yield compound **5a** (2.04 g, 77%) as an amorphous white. M.p. 109.3 °C [decomposition]. [$\alpha_{D}^{24.3}$] + 62° (*c* 0.20 g/100 mL; CHCl_3_). HRMS: *m*/*z* calcd for C_34_H_48_^79^BrNO_5_: 629.2710; found: 629.2701. Anal. Calcd. for C_34_H_48_BrNO_5_: C, 64.75; H, 7.67; Br, 12.67; N, 2.22; O, 12.68; found C, 65.10; H, 7.75; Br, 13.00; N, 2.35. ^1^H-NMR (CDCl_3_, 600 MHz): δ = 7.43 (s, 1H, NH), 7.38 (m, 4H, H-2′, H-3′, H-5′, H-6′), 5.03 (s, 1H, H-12(β)), 4.67 (m, 1H, H-3(β)), 2.37 (m, 1H, H-23), 2.18 (m, 1H, H-23′), 2.07 (s, 3H, CH_3_-26), 2.01 (s, 3H, CH_3_-28), 0.87 (s, 3H, CH_3_-19), 0.80 (d, 3H, *J*_21,20_ = 6.4, CH_3_-21), 0.69 (s, 3H, CH_3_-18). ^13^C-NMR (CDCl_3_, 150 MHz): δ = 171.55 (s, C-24), 170.51 (s, C-25), 170.44 (s, C-27), 136.96 (s, C-1′), 131.76 (d, C-3′), 131.76 (d, C-5′), 121.15 (d, C-2′), 121.15 (d, C-6′), 116.49 (s, C-4′), 75.82 (d, C-12), 74.08 (d, C-3), 49.27, 47.70, 44.90, 41.65, 35.50, 34.72, 34.56, 34.53, 34.24, 33.88, 32.10, 31.23, 27.25, 26.72, 26.48, 25.70, 25.52, 23.28, 22.93 (q, C-19), 21.36 (q, CH_3_-C(O)O), 21.30 (q, CH_3_-C(O)O), 17.53 (q, C-21), 12.33 (q, C-18).

#### 3.1.12. *N*-(4′-Bromophenyl)-3α,12α-dihydroxy-5β-cholan-24-amide (**5b**)

Crude product as an amorphous white solid (1.20 g, 81%) was prepared from diacetoxy derivative **5a** (1.75 g, 2.7 mmol) and KOH (0.91 g, 16.2 mmol) in MeOH (10 mL) according to the general procedure. An analytically pure sample of **5b** (1.17 g, 79%) was obtained by flash column chromatography (silica gel, 0.5–3% MeOH in CHCl_3_). M.p. 229.4 °C [decomposition]. [$\alpha_{D}^{26.8}$] + 25° (*c* 0.20 g/100 mL; CHCl_3_). HRMS: *m*/*z* calcd for C_30_H_44_^79^BrNO_3_: 545.2499; found: 545.2491. Anal. Calcd. for C_30_H_44_BrNO_3_: C, 65.92; H, 8.11; Br, 14.62; N, 2.56; O, 8.78; found C, 65.57; H, 7.90; Br, 14.62; N, 2.63. Anal. Calcd. for C_30_H_44_BrNO_3_: C, 65.92; H, 8.11; Br, 14.62; N, 2.56; O, 8.78; found C, 65.57; H, 7.90; Br, 14.62; N, 2.63. ^1^H-NMR (CDCl_3_, CD_3_OD, 400 MHz): δ = 9.00 (s, 1H, NH), 7.38 (d, 2H, *J* = 7.8, H-2′, H-6′), 7.29 (d, 2H, *J* = 7.8, H-3′, H-5′), 3.86 (s, 1H, H-12(β)), 3.47 (m, 1H, H-3(β)), 2.29 (m, 1H, H-23), 2.15 (m, 1H, H-23′), 0.90 (d, 3H, *J*_21,20_ = 5.7, CH_3_-21), 0.81 (s, 3H, CH_3_-19), 0.57 (s, 3H, CH_3_-18). ^13^C-NMR (CDCl_3_, CD_3_OD, 100 MHz): δ = 173.09 (s, C-24), 137.37 (s, C-1′), 131.42 (d, C-3′), 131.42 (d, C-5′), 121.14 (d, C-2′), 121.14 (d, C-6′), 115.99 (s, C-4′), 72.88 (d, C-12), 71.19 (d, C-3), 47.77, 46.23, 46.11, 41.77, 35.78, 35.65, 35.00, 34.97, 33.85, 33.29, 33.25, 31.10, 29.60, 28.20, 37.25, 26.85, 25.91, 23.45, 22.79 (q, C-19), 16.88 (q, C-21), 12.37 (q, C-18).

#### 3.1.13. *N*-(p-Tolyl)-3α,12α-diacetoxy-5β-cholan-24-amide (**6a**)

Crude product as an amorphous white solid (1.2 g, quantitative yield) was obtained from acid chloride intermediate (preparing by reaction diacetoxy derivative 3 (1.0 g, 2.1 mmol) and oxalyl chloride (1.1 mL, 12.6 mmol) in CH_2_Cl_2_ (10 mL)) and *p*-toluidine (0.27 g, 2.5 mmol) with addition of NEt_3_ (0.5 mL, 3.1 mmol) in CH_2_Cl_2_ (10 mL) according to the general procedure. The crude product was purified by flash column chromatography (silica gel, CHCl_3_) to yield compound **6a** (1.0 g, 84%) as an amorphous white. M.p. 94.8 °C [decomposition]. [$\alpha_{D}^{25.8}$] + 71° (*c* 0.20 g/100 mL; CHCl_3_). HRMS: *m*/*z* calcd for C_35_H_51_NO_5_: 565.3762; found: 565.3759. Anal. Calcd. for C_35_H_51_NO_5_: C, 74.30; H, 9.09; N, 2.48; O, 14.14; found C, 74.00; H, 8.72; N, 2.50. ^1^H-NMR (CDCl_3_, 600 MHz): δ = 7.65 (s, 1H, NH), 7.35 (d, 2H, *J* = 8.4, H-2′, H-6′), 7.05 (d, 2H, *J* = 8.3, H-3′, H-5′), 5.04 (s, 1H, H-12(β)), 4.65 (m, 1H, H-3(β)), 2.34 (m, 1H, H-23), 2.25 (s, 3H, CH_3_-7′), 2.15 (m, 1H, H-23′), 2.05 (c, 3H, CH_3_-26), 1.99 (s, 3H, CH_3_-28), 0.85 (s, 3H, CH_3_-19), 0.78 (d, 3H, *J*_21,20_ = 6.3, CH_3_-21), 0.67 (s, 3H, CH_3_-18). ^13^C-NMR (CDCl_3_, 150 MHz): δ = 171.49 (s, C-24), 170.43 (s, C-25), 170.36 (s, C-27), 135.39 (s, C-1′), 133.40 (s, C-4′), 129.18 (d, C-3′), 129.18 (d, C-5′), 119.70 (d, C-2′), 119.70 (d, C-6′), 75.79 (d, C-12), 74.02 (d, C-3), 49.21, 47.67, 44.81, 41.59, 35.44, 34.69, 34.49, 34.38, 34.17, 33.81, 32.04, 31.33, 27.18, 26.66, 26.42, 25.64, 25.44, 23.22, 22.86 (q, C-19), 21.27 (q, CH_3_-C(O)O), 21.21 (q, CH_3_-C(O)O), 20.64 (q, CH_3_-7′), 17.45 (q, C-21), 12.25 (q, C-18).

#### 3.1.14. *N*-(p-Tolyl)-3α,12α-dihydroxy-5β-cholan-24-amide (**6b**)

Crude product as an amorphous white solid (0.19 g, 98%) was prepared from diacetoxy derivative **6a** (0.23 g, 0.4 mmol) and KOH (0.13 g, 2.4 mmol) in MeOH (10 mL) according to the general procedure. An analytically pure sample of **6b** (0.14 g, 72%) was obtained by flash column chromatography (silica gel, 0–5% MeOH in CHCl_3_). M.p. 117.5 °C [decomposition]. [$\alpha_{D}^{26.3}$] + 33° (*c* 0.20 g/100 mL; CHCl_3_). HRMS: *m*/*z* calcd for C_31_H_47_NO_3_: 481.3551; found: 481.3553. Anal. Calcd. for C_31_H_47_NO_3_: C, 77.29; H, 9.83; N, 2.91; O, 9.96; found C, 77.60; H, 9.74; N, 2.99. ^1^H-NMR (CDCl_3_, 300 MHz): δ = 7.89 (s, 1H, NH), 7.40 (d, 2H, *J* = 8.2, H-2′, H-6′), 7.05 (d, 2H, *J* = 8.1, H-3′, H-5′), 3.96 (s, 1H, H-12(β)), 3.57 (m, 1H, H-3(β)), 2.90 (br s, 2H, OH), 2.26 (s, 3H, CH_3_-7′), 0.96 (d, 3H, *J*_21,20_ = 6.3, CH_3_-21), 0.87 (s, 3H, CH_3_-19), 0.64 (s, 3H, CH_3_-18). ^13^C-NMR (CDCl_3_, 75 MHz): δ = 172.18 (s, C-24), 135.61 (s, C-1′), 133.34 (d, C-4′), 129.23 (d, C-3′), 129.23 (d, C-5′), 119.70 (d, C-2′), 119.70 (d, C-6′), 73.13 (d, C-12), 71.65 (d, C-3), 47.80, 46.40, 46.37, 41.96, 36.19, 35.89, 35.18, 35.02, 34.05, 33.67, 33.43, 31.11, 30.43, 28.25, 27.46, 27.11, 26.07, 23.63, 22.99 (q, C-19), 20.71 (q, CH_3_-7′), 17.34 (q, C-21), 12.53 (q, C-18).

#### 3.1.15. *N*-(Pyridin-3-yl)-3α,12α-diacetoxy-5β-cholan-24-amide (**7a**)

Crude product as an amorphous white solid (1.34 g, quantitative yield) was obtained from acid chloride intermediate (preparing by reaction diacetoxy derivative 3 (1.0 g, 2.1 mmol) and oxalyl chloride (1.1 mL, 12.6 mmol) in CH_2_Cl_2_ (10 mL)) and 3-aminopyridine (0.24 g, 2.5 mmol) with addition of NEt_3_ (0.5 mL, 3.1 mmol) in CH_2_Cl_2_ (10 mL) according to the general procedure. The crude product was purified by flash column chromatography (silica gel, 0–5% MeOH in CHCl_3_) to yield compound **7a** (0.72 g, 62%) as an amorphous white. M.p. 161.2-161.7 °C. [$\alpha_{D}^{26}$] + 75° (*c* 0.20 g/100 mL; CHCl_3_). HRMS: *m*/*z* calcd for C_33_H_48_N_2_O_5_: 552.3558; found: 552.3563. Anal. Calcd. for C_33_H_48_N_2_O_5_: C, 71.71; H, 8.75; N, 5.07; O, 14.47; found C, 71.47; H, 8.52; N, 5.16. ^1^H-NMR (CDCl_3_, 600 MHz): δ = 8.54 (d, 1H, *J* = 2.3, H-2′), 8.30 (d, 1H, *J* = 4.6, H-4′), 8.23 (s, 1H, NH), 8.20 (d, 1H, *J* = 8.6, H-6′), 7.26 (dd, 1H, *J = J =* 4.1, H-5′), 5.07 (s, 1H, H-12(β)), 4.68 (m, 1H, H-3(β)), 2.44 (m, 1H, H-23), 2.25 (m, 1H, H-23′), 2.09 (s, 3H, CH_3_-26), 2.02 (s, 3H, CH_3_-28), 0.89 (s, 3H, CH_3_-19), 0.80 (d, 3H, *J*_21,20_ = 5.8, CH_3_-21), 0.70 (s, 3H, CH_3_-18). ^13^C-NMR (CDCl_3_, 150 MHz): δ = 172.30 (s, C-24), 170.53 (s, C-25), 170.50 (s, C-27), 144.68 (d, C-4′), 140.71 (d, C-6′), 135.07 (s, C-3′), 127.06 (d, C-2′), 123.64 (d, C-5′), 75.80 (d, C-12), 74.07 (d, C-3), 49.22, 47.65, 44.86, 41.60, 35.45, 34.71, 34.28, 34.20, 33.85, 32.06, 31.16, 29.53, 27.23, 26.68, 26.45, 25.67, 25.50, 23.26, 22.91 (q, C-19), 21.36 (q, CH_3_-C(O)O), 21.30 (q, CH_3_-C(O)O), 17.49 (q, C-21), 12.30 (q, C-18).

#### 3.1.16. *N*-(Pyridin-3-yl)-3α,12α-dihydroxy-5β-cholan-24-amide (**7b**)

Crude product as an amorphous white solid (0.44 g, 88%) was prepared from diacetoxy derivative **7a** (0.59 g, 1.1 mmol) and KOH (0.37 g, 6.6 mmol) in MeOH (10 mL) according to the general procedure. An analytically pure sample of **7b** (0.22 g, 44%) was obtained by flash column chromatography (silica gel, 1–5% MeOH in CHCl_3_). M.p. 114.6 °C [decomposition]. [$\alpha_{D}^{27.1}$] + 35° (*c* 0.20 g/100 mL; CH_3_OH). HRMS: *m*/*z* calcd for C_29_H_44_N_2_O_3_: 468.3347; found: 468.3344. Anal. Calcd. for C_29_H_44_N_2_O_3_: C, 74.32; H, 9.46; N, 5.98; O, 10.24; found C, 73.98; H, 9.44; N, 5.70. ^1^H-NMR (CDCl_3_, MeOH, 400 MHz): δ = 9.53 (s, 1H, NH), 8.45 (s, 1H, H-2′), 8.24 (d, 1H, *J* = 7.9, H-4′), 8.14 (d, 1H, *J* = 4.2, H-6′), 7.23 (m, 1H, H-5′), 3.88 (s, 1H, H-12(β)), 3.48 (m, 1H, H-3(β)), 2.36 (m, 1H, H-23), 2.22 (m, 1H, H-23′), 0.93 (d, 3H, *J*_21,20_ = 5.8, CH_3_-21), 0.82 (s, 3H, CH_3_-19), 0.59 (s, 3H, CH_3_-18). ^13^C-NMR (CDCl_3_, MeOH, 100 MHz): δ = 173.77 (s, C-24), 143.07 (d, C-4′), 139.74 (d, C-6′), 135.97 (s, C-3′), 127.69 (d, C-2′), 123.87 (d, C-5′), 72.91 (d, C-12), 71.19 (d, C-3), 47.84, 46.20, 46.14, 41.79, 35.77, 35.68, 35.00, 34.98, 33.88, 33.27, 33.10, 31.04, 29.62, 28.24, 27.28, 26.85, 25.93, 23.48, 22.82 (q, C-19), 16.89 (q, C-21), 12.41 (q, C-18).

#### 3.1.17. *N*-(1′-Adamantyl)-3α,12α-diacetoxy-5β-cholan-24-amide (**8a**)

Crude product as an amorphous white solid (0.73 g, 95%) was obtained from acid chloride intermediate (preparing by reaction diacetoxy derivative 3 (0.60 g, 1.3 mmol) and oxalyl chloride (0.6 mL, 7.8 mmol) in CH_2_Cl_2_ (10 mL)) and 1-aminoadamantane (prepared in situ from of 1-aminoadamantane hydrochloride (0.26 g, 1.4 mmol) and NEt_3_ (0.02 mL, 0.14 mmol)), with addition of NEt_3_ (0.3 mL, 2.0 mmol) in CH_2_Cl_2_ (10 mL) according to the general procedure. The crude product was purified by flash column chromatography (silica gel, CHCl_3_) to yield compound **8a** (0.36 g, 47%) as an amorphous white. M.p. 120.7 °C. [decomposition]. [$\alpha_{D}^{26.1}$] + 68° (*c* 0.20 g/100 mL; CHCl_3_). HRMS: *m*/*z* calcd for C_38_H_59_NO_5_: 609.4388; found: 609.4383. Anal. Calcd. for C_38_H_59_NO_5_: C, 74.84; H, 9.75; N, 2.30; O, 13.12; found C, 74.76; H, 9.83; N, 2.41. ^1^H-NMR (CDCl_3_, 600 MHz): δ = 5.07 (s, 1H, H-12(β)), 5.03 (dd, 1H, *J* = 5.0, NH), 4.66 (m, 1H, H-3(β)), 2.05 (s, 3H, CH_3_-26), 2.02 (br s, 3H, H-3′, H-5′, H-8′), 1.99 (s, 3H, CH_3_-28), 1.94 (s, 6H, CH_2_-2′, CH_2_-6′, CH_2_-9′), 1.63 (m, 6H, CH_2_-4′, CH_2_-7′, CH_2_-10′), 0.86 (s, 3H, CH_3_-19), 0.76 (d, 3H, *J*_21,20_ = 6.5, CH_3_-21), 0.68 (s, 3H, CH_3_-18). ^13^C-NMR (CDCl_3_, 150 MHz): δ = 172.31 (s, C-24), 170.39 (s, C-25), 170.31 (s, C-27), 75.80 (d, C-12), 74.04 (d, C-3), 51.56, 49.27, 47.67, 44.87, 41.68, 41.55 (t, C-2′, C-6′, C-9′), 36.22 (t, C-4′, C-7′, C-10′), 35.53, 34.64, 34.57, 34.53, 34.25, 33.88, 32.11, 31.49, 29.29 (d, C-3′, C-5′, C-8′), 27.22, 26.74, 26.47, 25.71, 25.49, 23.28, 22.91 (q, C-19), 21.30 (q, CH_3_-C(O)O), 21.23 (q, CH_3_-C(O)O), 17.53 (q, C-21), 12.29 (q, C-18).

#### 3.1.18. *N*-(1′-Adamantyl)-3α,12α-dihidroxy-5β-cholan-24-amide (**8b**)

Crude product as an amorphous white solid (0.17 g, quantitative yield) was prepared from diacetoxy derivative **8a** (0.20 g, 0.3 mmol) and KOH (0.10 g, 1.8 mmol) in MeOH (5 mL) according to the general procedure. An analytically pure sample of **8b** (0.12 g, 71%) was obtained by flash column chromatography (silica gel, 1–5% MeOH in CHCl_3_). M.p. 300.8 °C [decomposition]. [$\alpha_{D}^{27}$] +23° (*c* 0.20 g/100 mL; CH_3_OH). HRMS: *m*/*z* calcd for C_34_H_55_NO_3_: 525.4177; found: 525.4173. Anal. Calcd. for C_34_H_55_NO_3_: C, 77.66; H, 10.54; N, 2.66; O, 9.13; found C, 77.92; H, 10.29; N, 2.79. ^1^H-NMR (CDCl_3_, CD_3_OD, 400 MHz): δ = 3.79 (s, 1H, H-12(β)), 3.38 (m, 1H, H-3(β)), 1.90 (br s, 3H, H-3′, H-5′, H-8′), 1.82 (s, 6H, CH_2_-2′, CH_2_-6′, CH_2_-9′), 1.51 (s, 6H, CH_2_-4′, CH_2_-7′, CH_2_-10′), 0.81 (d, 3H, *J*_21,20_ = 6.3, CH_3_-21), 0.74 (s, 3H, CH_3_-19), 0.51 (s, 3H, CH_3_-18). ^13^C-NMR (CDCl_3_, CD_3_OD, 100 MHz): δ = 173.90 (s, C-24), 72.67 (d, C-12), 71.00 (d, C-3), 51.41, 47.64, 46.39, 46.01, 41.72, 40.95 (t, C-2′, C-6′, C-9′), 35.91 (t, C-4′, C-7′, C-10′), 35.57, 35.54, 34.97, 34.89, 34.74, 33.12, 31.45, 29.38, 29.01 (d, C-3′, C-5′, C-8′), 28.09, 27.19, 26.74, 25.80, 23.34, 22.64 (q, C-19), 16.66 (q, C-21), 12.23 (q, C-18).

#### 3.1.19. *N*-(2′′-(3′,5′-Di-tret-buthyl-4′-hydroxyphenyl)-ethyl)-3α,12α-diacetoxy-5β-cholan-24-amide (**9a**)

Crude product as an amorphous white solid (0.29 g, 90%) was obtained from acid chloride intermediate (preparing by reaction diacetoxy derivative 3 (0.20 g, 0.40 mmol) and oxalyl chloride (0.2 mL, 2.5 mmol) in CH_2_Cl_2_ (10 mL)) and 2-(3′,5′-di*-tret*-buthyl-4′-hydroxyphenyl)ethan-1-amine (0.11 g, 0.44 mmol) with addition of NEt_3_ (0.1 mL, 0.66 mmol) in CH_2_Cl_2_ (10 mL) according to the general procedure. The crude product was purified by flash column chromatography (silica gel, 0–2% MEOH in CHCl_3_) to yield compound **9a** (0.09 g, 28%) as an amorphous white. M.p. 98.7 °C. [decomposition]. [$\alpha_{D}^{23.5}$] +66° (*c* 0.20 g/100 mL; CH_3_OH). HRMS: *m*/*z* calcd for C_44_H_69_O_6_N_1_: 707.5119; found: 707.5122. Anal. Calcd. for C_44_H_69_NO_6_: C, 74.64; H, 9.82; N, 1.98; O, 13.56; found C, 74.38; H, 9.86; N, 2.11. ^1^H-NMR (CDCl_3_, 300 MHz): δ = 6.94 (s, 2H, H-2′, H-6′), 5.48 (m, 1H, NH), 5.09 (s, 1H, OH), 5.04 (s, 1H, H-12(β)), 4.67 (m, 1H, H-3(β)), 3.43 (m, 2H, CH_2_-16′), 2.69 (m, 2H, CH_2_-15′), 2.06 (s, 3H, CH_3_-26), 2.00 (s, 3H, CH_3_-28), 0.87 (s, 3H, CH_3_-19), 0.77 (d, 3H, *J*_21,20_ = 6.1, CH_3_-21), 0.69 (s, 3H, CH_3_-18). ^13^C-NMR (CDCl_3_, 75 MHz): δ = 173.03 (s, C-24), 170.40 (s, C-25), 170.30 (s, C-27), 152.21 (s, C-4′), 135.94 (s, C-3′), 135.94 (s, C-5′), 129.31 (s, C-1′), 125.18 (d, C-2′), 125.18 (d, C-6′), 75.77 (d, C-12), 74.04 (d, C-3), 49.26, 47.58, 44.86, 41.67, 40.79, 35.52, 35.49, 34.74, 34.57, 34.24, 34.14, 33.88, 33.59, 32.11, 31.53, 30.17 (q, C(CH_3_)_3_), 27.25, 26.73, 26.48, 25.70, 25.49, 23.26, 22.92 (q, C-19), 21.31 (q, CH_3_-C(O)O), 21.23 (q, CH_3_-C(O)O), 17.49 (q, C-21), 12.28 (q, C-18).

#### 3.1.20. *N*-(3′,3′-Diethylaminopropyl)-3α,12α-diacetoxy-5β-cholan-24-amide (**10a**)

Crude product as an amorphous yellow (2.2 g, 92%) was obtained from acid chloride intermediate (preparing by reaction diacetoxy derivative 3 (2.0 g, 4.2 mmol) and oxalyl chloride (2.1 mL, 25.2 mmol) in CH_2_Cl_2_ (10 mL)) and *N*^1^,*N*^1^-diethylaminopropan-1,3-diamine (0.65 g, 5.0 mmol) with addition of NEt_3_ (0.9 mL, 6.3 mmol) in CH_2_Cl_2_ (20 mL) according to the general procedure. The crude product was purified by flash column chromatography (silica gel, 0–20% MeOH in CHCl_3_) to yield compound **10a** (1.70 g, 67%) as an amorphous yellow. M.p. 45.8 °C [decomposition]. [$\alpha_{D}^{24.8}$] + 75° (*c* 0.20 g/100 mL; CHCl_3_). HRMS: *m*/*z* calcd for C_35_H_60_N_2_O_5_: 588.4497; found: 588.4495. Anal. Calcd. for C_35_H_60_N_2_O_5_: C, 71.39; H, 10.27; N, 4.76; O, 13.59; found C, 71.44; H, 10.23; N, 4.79. ^1^H-NMR (CDCl_3_, 400 MHz): δ = 7.44 (m, 1H, NH), 4.99 (s, 1H, H-12(β)), 4.60 (m, 1H, H-3(β)), 3.23 (ddd, 2H, *J = J = J =* 5.6, CH_2_-1′), 2.41 (ddd, 6H, *J = J = J =* 7.0, CH_2_-3′, CH_2_-4′, CH_2_-6′), 2.00 (s, 3H, CH_3_-26), 1.94 (s, 3H, CH_3_-28), 0.81 (s, 3H, CH_3_-19), 0.71 (d, 3H, *J*_21,20_ = 6.2, CH_3_-21), 0.63 (s, 3H, CH_3_-18). ^13^C-NMR (CDCl_3_, 100 MHz): δ = 172.83 (s, C-24), 170.27 (s, C-25), 170.20 (s, C-27), 75.64 (d, C-12), 73.89 (d, C-3), 52.49, 49.14, 47.44, 46.54 (t, C-4′), 46.54 (t, C-6′), 44.69, 41.52, 39.72, 35.35, 34.61, 34.42, 34.09, 33.73, 33.49, 31.94, 31.55, 27.10, 26.59, 26.33, 25.56, 25.35, 25.12, 23.15, 22.80 (q, C-19), 21.20 (q, CH_3_-C(O)O), 21.10 (q, CH_3_-C(O)O), 17.32 (q, C-21), 12.13 (q, C-18), 11.60 (q, C-5′), 11.60 (q, C-7′).

#### 3.1.21. *N*-(3′-Morpholinopropyl)-3α,12α-diacetoxy-5β-cholan-24-amide (**11a**).

Crude product as an amorphous solid (2.5 g, quantitative yield) was obtained from acid chloride intermediate (preparing by reaction diacetoxy derivative 3 (2.0 g, 4.2 mmol) and oxalyl chloride (2.1 mL, 25.2 mmol) in CH_2_Cl_2_ (10 mL)) and 3-morpholinopropan-1-amine (0.72 g, 5.0 mmol) with addition of NEt_3_ (0.9 mL, 6.3 mmol) in CH_2_Cl_2_ (20 mL) according to the general procedure. The crude product was purified by successive flash column chromatographies: (1) silica gel, 0–1% MeOH in CHCl_3_; (2) silica gel, 15–40% AcOEt in *n*-hexane, to yield compound **11a** (1.75 g, 69%) as an amorphous white solid. M.p. 61.0 °C [decomposition]. [$\alpha_{D}^{25}$] + 78° (*c* 0.20 g/100 mL; CHCl_3_). HRMS: *m*/*z* calcd for C_35_H_58_N_2_O_6_: 602.4289; found: 602.4287. Anal. Calcd. for C_35_H_58_N_2_O_6_: C, 69.73; H, 9.70; N, 4.65; O, 15.92 found C, 69.75; H, 9.73; N, 4.62; ^1^H-NMR (CDCl_3_, 400 MHz): δ = 6.81 (dd, 1H, *J* = 5.0, NH), 4.98 (s, 1H, H-12(β)), 4.60 (m, 1H, H-3(β)), 3.61 (dd, 4H, *J = J =* 4.2, CH_2_-5′, CH_2_-6′), 3.23 (ddd, 2H, *J = J = J =* 5.9, CH_2_-1′), 2.36 (m, 6H, CH_2_-3′, CH_2_-4′, CH_2_-7′), 2.00 (s, 3H, CH_3_-26), 1.94 (s, 3H, CH_3_-28), 0.81 (s, 3H, CH_3_-19), 0.72 (d, 3H, *J*_21,20_ = 6.3, CH_3_-21), 0.63 (s, 3H, CH_3_-18). ^13^C-NMR (CDCl_3_, 100 MHz): δ = 172.92 (s, C-24), 170.27 (s, C-25), 170.19 (s, C-27), 75.59 (d, C-12), 73.87 (d, C-3), 66.72 (t, C-5′, C-6′), 57.47 (t, C-3′), 53.40 (t, C-4′, C-7′), 49.11, 47.46, 44.69, 41.50, 38.86 (t, C-1′), 35.33, 34.60, 34.41, 34.06, 33.71, 33.50, 31.92, 31.49, 27.12, 26.69, 26.57, 26.31, 25.54, 25.34, 24.75, 23.13, 22.80 (q, C-19), 21.20 (q, CH_3_-C(O)O), 21.09 (q, CH_3_-C(O)O), 17.34 (q, C-21), 12.15 (q, C-18).

### 3.2. Tdp1 Assay 

The recombinant Tdp1 was purified to homogeneity by chromatography on Ni-chelating resin and phosphocellulose P11 as described [42,43] using the plasmid pET 16B-Tdp1 kindly provided by Dr. K.W. Caldecott (University of Sussex, United Kingdom). 

Real-Time Detection of Tdp1 Activity. The Tdp1 activity measurements were carried out as described [25]. Briefly, Tdp1-biosensor with a final concentration of 50 nM was incubated in a volume of 200 μL containing a Tdp1 buffer (50 mM Tris-HCl pH8.0, 50 mM NaCl, 7 mM β-mercaptoethanol) supplemented with purified 1.3 nM Tdp1 and various concentrations of the inhibitor in a 1.5% DMSO solution. Fluorescence measurements (Ex_485_/Em_520_ nm) were carried out during the linear phase of reaction (from 0 to 8 min) every 55 s. The reactions were incubated at a constant temperature of 26 °C in a POLARstar OPTIMA fluorimeter, BMG LABTECH, GmbH. The influence of compounds was evaluated by comparing the fluorescence increase rate in the presence of compounds to that of the DMSO control wells. The data were imported into the MARS Data Analysis 2.0 program (BMG LABTECH), and IC_50_ values (concentration of a compound required to reduce the enzyme activity by 50%) were calculated. Tdp1-biosensor 5′-(5,6 FAM-aac gtc agg gtc ttc c-BHQ1)-3′ was synthesized in the Laboratory of Biomedical Chemistry, Institute of Chemical Biology and Fundamental Medicine, Novosibirsk, Russia.

IC_50_ values are listed in Table 1 and Table 2.

### 3.3. Analysis of Cytotoxicity

Cytotoxicity analysis of the compounds was carried out on the cell lines MCF-7 (human breast adenocarcinoma) and HCT-116 (human colon carcinoma). Compound-induced cell death was assessed using a standard MTT test [44]. MCF-7 and HCT-116 cells (~2000 cells per well) were incubated for 24 h at 37 °C in an IMDM medium containing 10% (*v*/*v*) fetal bovine serum, 40 µg/mL gentamicin, 100 U/mL penicillin, 0.1 mg/mL streptomycin, and 0.25 µg/mL amphotericin in a humidified atmosphere (5% CO_2_). After the formation of a 50% monolayer, the test preparations in DMSO were added to the culture medium (the volume of the added reagents was 1/100 of the total volume of the culture medium, the volume of DMSO was 1% of the final volume), and the growth of the cell culture was observed for three days. Control cells were grown in the presence of 1% DMSO.

The toxicity of the compounds was absent or was insignificant throughout the range of concentrations studied (up to 100 μM).

### 3.4. Virtual Screening and Molecular Modelling

The compounds were screened/docked to the crystal structure of Tdp1 (PDB ID: 1MU7, resolution 2.0 Å) [32], which was obtained from the Protein Data Bank (PDB) [45,46]. The SciGress FJ 2.6 program was used to prepare the crystal structure for docking [47], i.e., hydrogen atoms were added, and the co-crystallized tungsten(VI)ion was removed as well as the crystallographic water molecules. The SciGress software suite was also used to build the inhibitors and the MM2 force field was used to optimize the structures [48]. The center of the binding pocket was defined as the position of the hydrogen atom of His263 and the ND1 nitrogen formed a coordination bond with the tungsten ion (*x* = 8.312, *y* = 12.660, *z* = 35.452) with 10 Å radius. For the initial screen, 30% search efficiency was used with ten runs per compound. For the second phase (re-dock) and the molecular modelling, 100% efficiency was used in conjunction with fifty docking runs. The basic amino acids lysine and arginine were defined as protonated. Furthermore, aspartic and glutamic acids were assumed to be deprotonated. The GoldScore (GS) [49], ChemScore (CS) [50,51], Chem Piecewise Linear Potential (ChemPLP) [52], and Astex Statistical Potential (ASP) [53] scoring functions were implemented to validate the predicted binding modes and relative energies of the ligands using the GOLD v5.4 software suite [49]. The virtual screen was conducted with of 9.2 × 10^3^ molecular entities obtained from the InterBioScreen natural product collection. The Marvin Sketch software package was used to calculate the molecular descriptors of the compounds [54]. 

## 4. Conclusions

Thus, the amides obtained from deoxycholic acid and containing tryptamine (compound **1a**–**3a**), *p*-bromoaniline (compound **5a**), 1-aminoadamantane (compound **8a**) and 4-(2-aminoethyl)-2,6-bis-*t*-butylphenol (compound **9a**) fragments demonstrated high inhibition activity against Tdp1 with IC_50_ values in the range 0.29–0.47 μM. It should also be noted that these compounds have a more pronounced activity than the reference substance furamidine. The compounds demonstrated low cytotoxicity which may simplify their use as a component of “cocktails” with known anticancer drugs. Thus, the compounds can be considered as a basis for the development of new promising agents for the combined chemotherapy of oncological diseases.

Obviously, the use of metabolically unstable acetoxy group makes the successful transition to in vivo experiments unlikely. Therefore, the purpose of our further research will be the modification of the steroid scaffold by metabolically stable functional groups and the study of their effect on inhibitory activity against Tdp1.

## Figures and Tables

**Table 1:** Tdp1 Inhibitory activity of compounds **1a**,**b**, **2a**,**b** and **3a**–**d**.

Compound	IC_50_^1^ μM	Compound	IC_50_^1^ μM
**1a**	0.32 ± 0.11	**1b**	2.65 ± 0.3
**2a**	0.38 ± 0.12	**2b**	2.6 ± 0.4
**3a**	0.65 ± 0.16	**3b**	2.7 ± 0.2
**3c**	0.95 ± 0.05	**3d**	0.48 ± 0.04
**Fur** ^2^	1.2 ± 0.3		

^1^ **IC_50_** is the concentration that inhibits the activity of the enzyme by 50%. ^2^ **Fur** is furamidine (**A**) (Figure 1).

**Table 2:** Tdp1 Inhibitory activity of compounds **4a**,**b**–**8a**,**b** and **9a**–**11a**.

Compound	IC_50_^1^ μM	Compound	IC_50_^1^ μM
**4a**	0.43 ± 0.13	**4b**	6.7 ± 0.7
**5a**	0.42 ± 0.01	**5b**	1.3 ± 0.2
**6a**	1.00 ± 0.05	**6b**	7.6 ± 3.9
**7a**	4.08 ± 0.08	**7b**	>15
**8a**	0.47 ± 0.08	**8b**	2.3 ± 0.4
**9a**	0.29 ± 0.12		
**10a**	>15		
**11a**	>15		
**Fur** ^2^	1.2 ± 0.3		

^1^ **IC_50_** is the concentration that inhibits the activity of the enzyme by 50%. ^2^ **Fur** is furamidine (**A**) (Figure 1).

## Supplementary Materials

Supplementary materials are available online: virtual screening & molecular modeling data and copies of ^1^H-NMR and ^13^C-NMR spectra of all compounds.
