# Supplementary material for: Novel Semisynthetic Derivatives of Bile Acids as Effective Tyrosyl-DNA Phosphodiesterase 1 Inhibitors
Source: Molecules. 2018 Mar 17;23(3):679. doi: 10.3390/molecules23030679 (PMC6017735; doi:10.3390/molecules23030679)

Novel Semisynthetic Derivatives of Bile Acids as Effective Tyrosyl-DNA Phosphodiesterase 1 Inhibitors

Oksana V. Salomatina[1], Irina I. Popadyuk[1], Alexandra L. Zakharenko[2], Olga D. Zakharova[2], Dmitriy S. Fadeev[1], Nina I. Komarova[1], Jóhannes Reynisson[3], H. John Arabshahi[3], Raina Chand[3], Konstantin P. Volcho[1,4], Nariman F. Salakhutdinov[1,4], Olga I. Lavrik[2,4]

[1] *N.N. Vorozhtsov Novosibirsk Institute of Organic Chemistry, SB RAS, 630090, Russia, Novosibirsk, acad. Lavrentjev ave. 9; ana@nioch.nsc.ru (O.V.S.), popadyuk@nioch.nsc.ru (I.I.P.), dsf@nioch.nsc.ru (D.S.F.), komar@nioch.nsc.ru (N.I.K)*
[2] *Novosibirsk Institute of Chemical Biology and Fundamental Medicine, SB RAS, 630090, Russia, Novosibirsk, acad. Lavrentjev ave. 8; sashaz@niboch.nsc.ru (A.L.Z), isar@niboch.nsc.ru (O.D.Z.)*
[3] *School of Chemical Sciences, University of Auckland, New Zealand. j.reynisson@auckland.ac.nz (J.R.), j.arabshahi@auckland.ac.nz (H.J.A.), rcha387@aucklanduni.ac.nz (R.Ch.)*
[4] *Novosibirsk State University, 630090, Russia, Novosibirsk, Pirogova str. 2; anvar@nioch.nsc.ru (N.F.S.) lavrik@niboch.nsc.ru (O.I.L.)*
* *Correspondence: volcho@nioch.nsc.ru; Tel.:* +7 383 3308870

ELECTRONIC SUPPORTING INFORMATION

Virtual screening & molecular modeling data……………………………………………………2
NMR ^{1}H and ^{13}C spectra of new compounds…………………………………………………5

Table S1. The scores predicted by the four scoring functions for compounds **1a,b, 2a,b, 3a-d, 4a,b-8a,b** and **9a-11a** docked in Tdp1.

**Compound**	**Scoring Function**				**IC_{50}**
	**PLP**	**GS**	**CS**	**ASP**	
**1a**	71.8	56.5	32.7	36.0	0.32±0.11
**2a**	70.4	58.6	26.7	31.6	0.38±0.12
**3a**	80.8	60.8	33.9	35.8	0.65±0.16
**3c**	71.9	49.7	22.9	35.4	0.95±0.05
**Fur**	45.3	47.6	27.1	33.3	1.23±0.33
**1b**	75.2	44.6	36.3	33.6	2.65±0.30
**2b**	74.8	59.5	37.4	35.7	2.6±0.4
**3b**	80.0	63.5	33.5	34.1	2.7±0.2
**3d**	72.7	67.9	34.1	37.4	0.48±0.04
**4a**	41.5	28.2	26.7	28.5	0.43±0.13
**4b**	65.9	41.9	31.3	28.2	6.7±0.7
**5a**	62.1	53.9	31.2	27.3	0.42±0.01
**5b**	66.4	49.7	31.5	27.5	1.3±0.2
**6a**	64.7	40.3	29.5	27.0	1.00±0.05
**6b**	66.5	45.8	32.4	28.2	7.6±3.9
**7a**	63.3	59.4	29.8	30.0	4.08±0.08
**7b**	63.4	50.1	29.6	32.5	>15
**8a**	64.5	60.2	30.8	26.8	0.47±0.08
**8b**	63.5	47.3	33.0	27.2	2.3±0.4
**9a**	69.4	59.1	31.5	35.0	0.29±0.12
**10a**	56.9	56.2	25.6	26.8	>15
**11a**	56.5	61.9	22.1	28.6	>15

Table S2. The calculated molecular descriptors for the bile acid derivatives.

Compound	Molecular Descriptor					
	**Mol. weight**	**Log P**	**TPSA**	**H bond donor**	**H bond acceptor**	**Rotatable bond**
**1a**	618.9	5.5	97.5	2	3	11
**2a**	618.9	5.5	97.5	2	3	11
**3a**	618.9	5.3	97.5	2	3	11
**3c**	576.8	5.2	91.4	3	3	9
**Fur**	304.4	2.9	112.9	4	4	4
**1b**	534.8	5.0	85.4	4	3	7
**2b**	534.8	5.0	85.4	4	3	7
**3b**	534.8	5.0	85.4	4	3	7
**3d**	576.8	5.2	91.4	3	3	9
**4a**	551.8	5.7	81.7	1	3	9
**4b**	467.7	5.4	69.6	3	3	5
**5a**	630.6	6.5	81.7	1	3	9
**5b**	546.6	6.2	69.5	3	3	5
**6a**	565.8	6.1	81.7	1	3	9
**6b**	481.7	5.9	69.6	3	3	5
**7a**	552.8	4.4	94.6	1	4	9
**7b**	468.7	4.1	82.5	3	4	5
**8a**	609.9	5.7	81.7	1	3	9
**8b**	525.8	5.4	69.6	3	3	5
**9a**	708.0	9.0	101.9	2	4	13
**10a**	588.9	4.7	84.9	1	4	14
**11a**	589.8	4.0	94.2	1	5	12

Table S3. Criteria of lead-like, drug-like and known drug space (KDS) in terms of molecular descriptors.

	**Lead-like Space**	**Drug-like Space**	**Known Drug Space**
Molecular weight (g mol^{-1})	300	500	800
Lipophilicity (Log P)	3	5	6.5
Hydrogen bond donors (HD)	3	5	7
Hydrogen bond acceptors (HA)	3	10	15
Polar surface area ($Å^2$) (PSA)	60	140	180
Rotatable bonds (RB)	3	10	17

From: Zhu, F; Logan, G.; Reynisson J. Wine Compounds as a Source for HTS Screening Collections. A Feasibility Study**.** *Mol. Inf.*, **2012**, *31*, 847 – 855**,** DOI:10.1002/minf.201200103

Spectra of Compound **1**, ^{1}H NMR, 300MHz, $CDCl_3$ (bottom); ^{13}C NMR, JMOD, 75MHz, $CDCl_3$ (top)

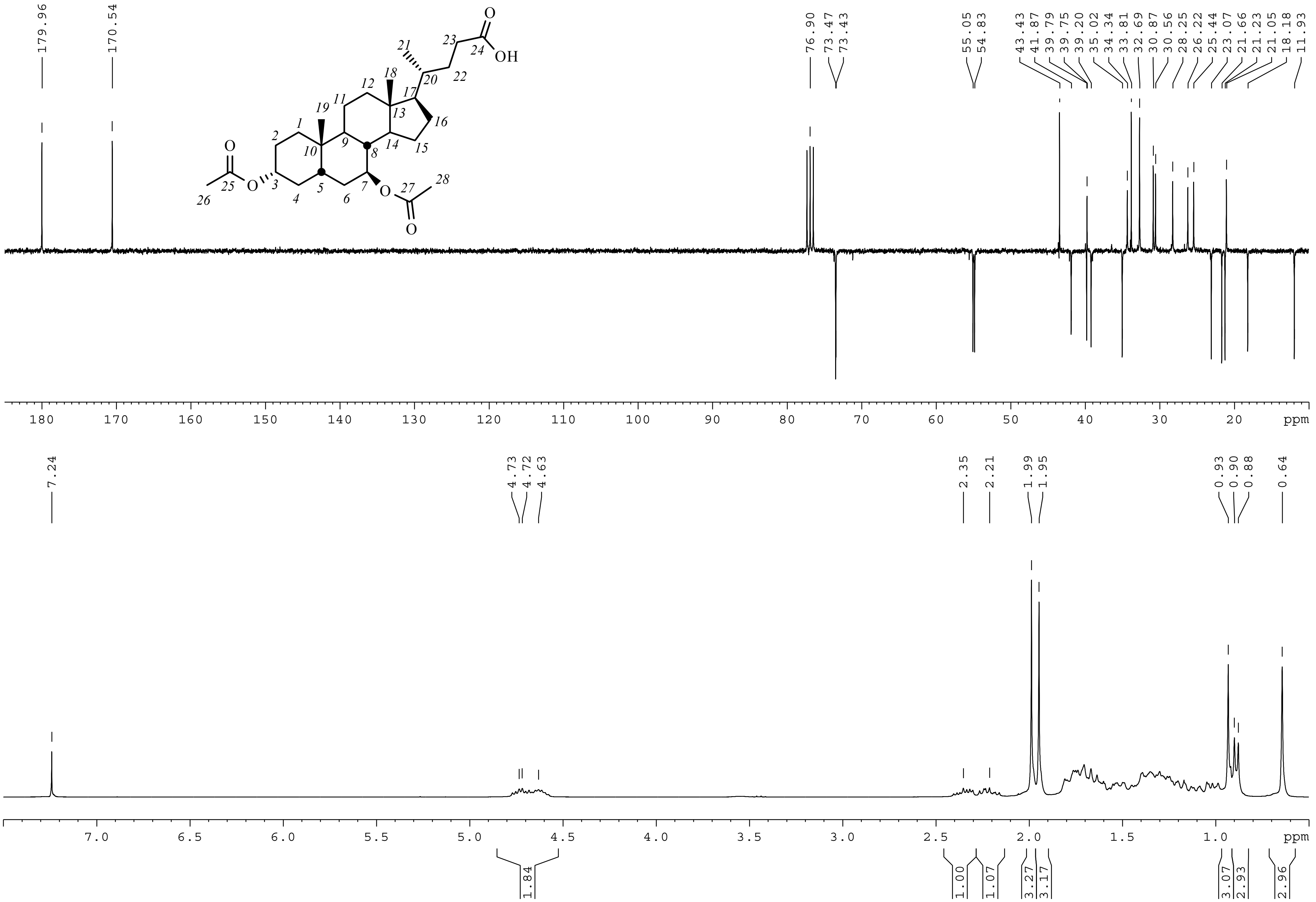

Spectra of Compound **2**, ^{1}H NMR, 500MHz, CDCl$_3$ (bottom); ^{13}C NMR, JMOD, 125MHz, CDCl$_3$ (top)

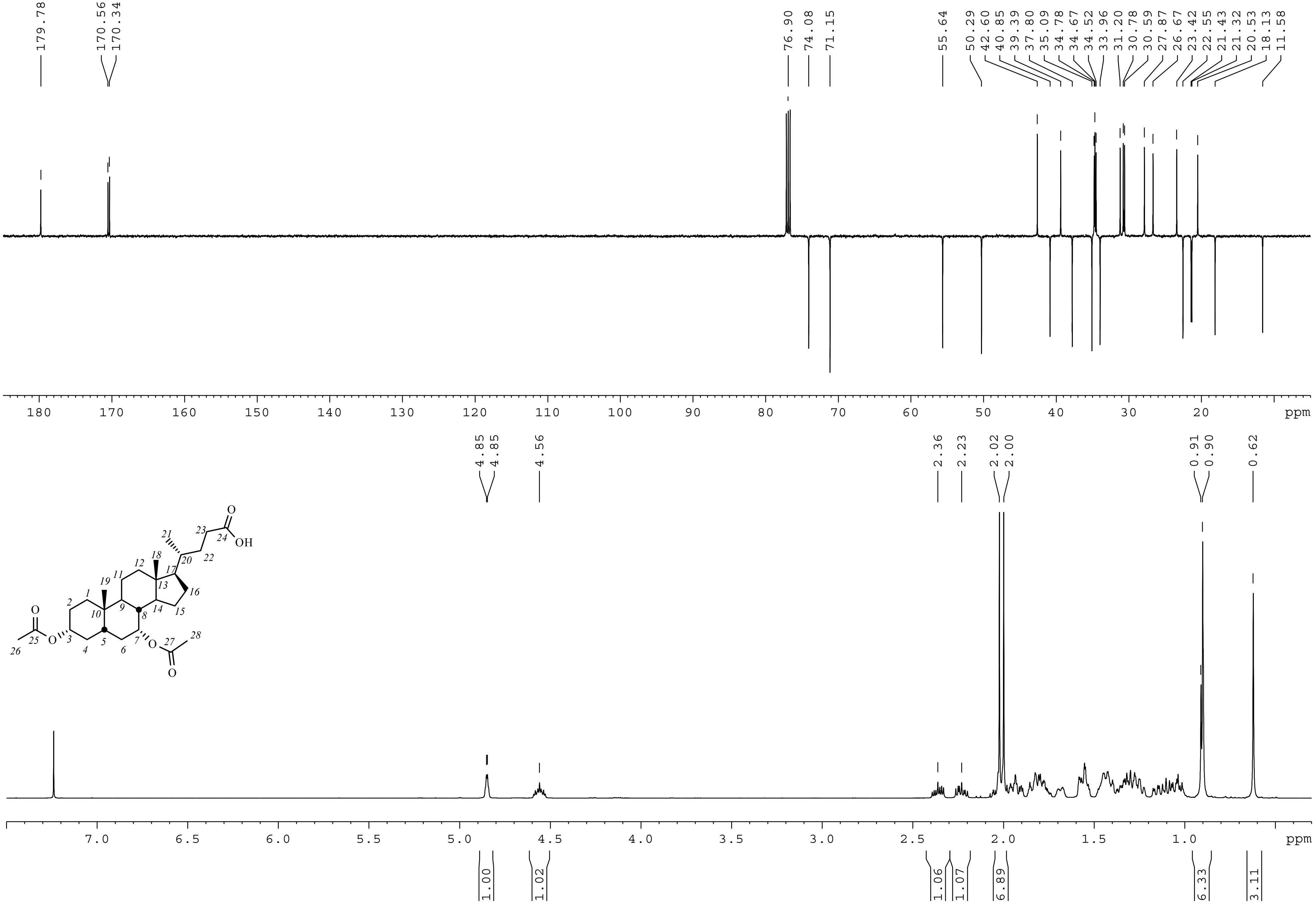

Spectra of Compound **3**, ^{1}H NMR, 300MHz, $CDCl_3$ (bottom); ^{13}C NMR, JMOD, 75MHz, $CDCl_3$ (top)

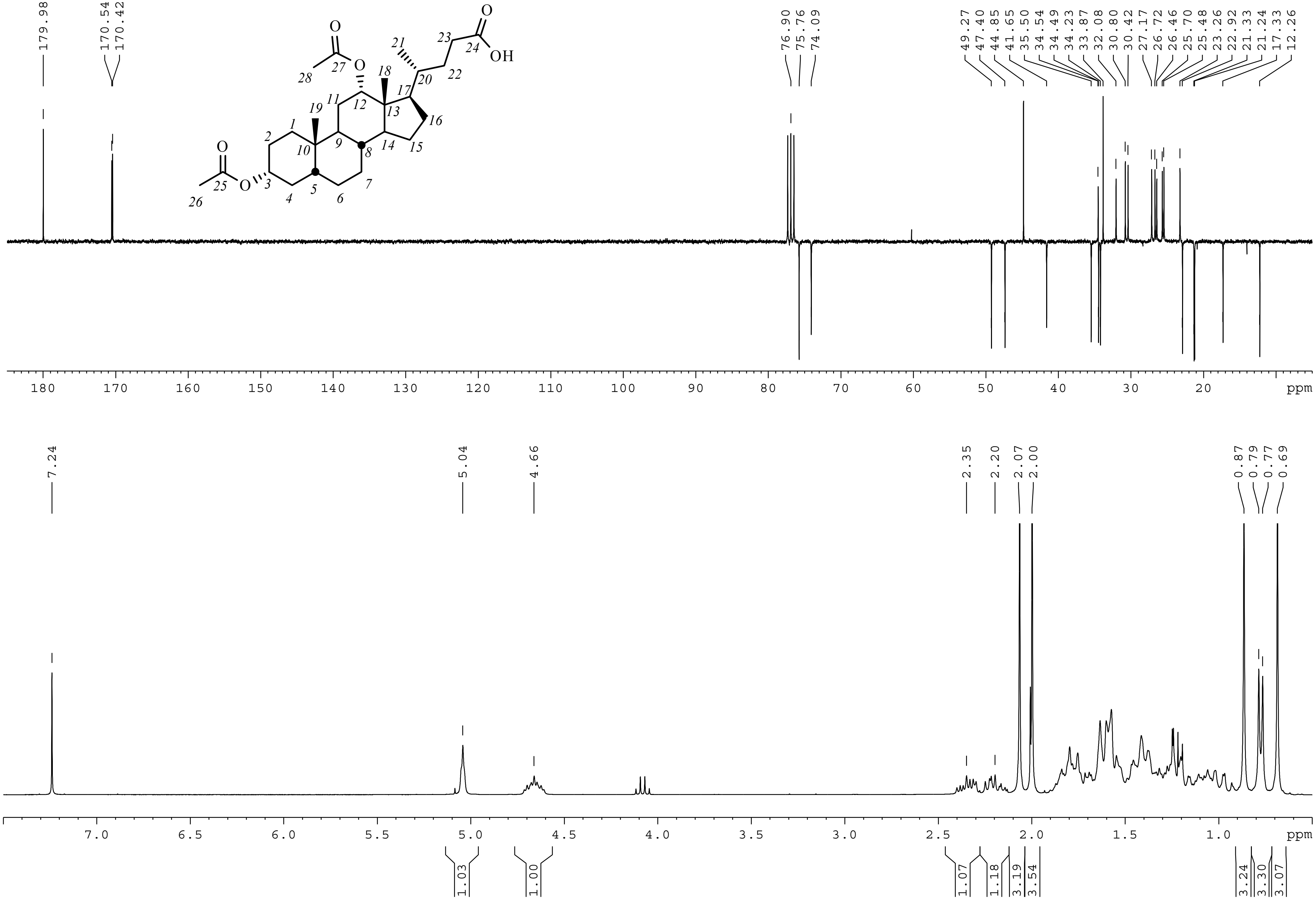

Spectra of Compound **1a**, ^{1}H NMR, 400MHz, $CDCl_3$ (bottom); ^{13}C NMR, JMOD, 125MHz, $CDCl_3$ (top)

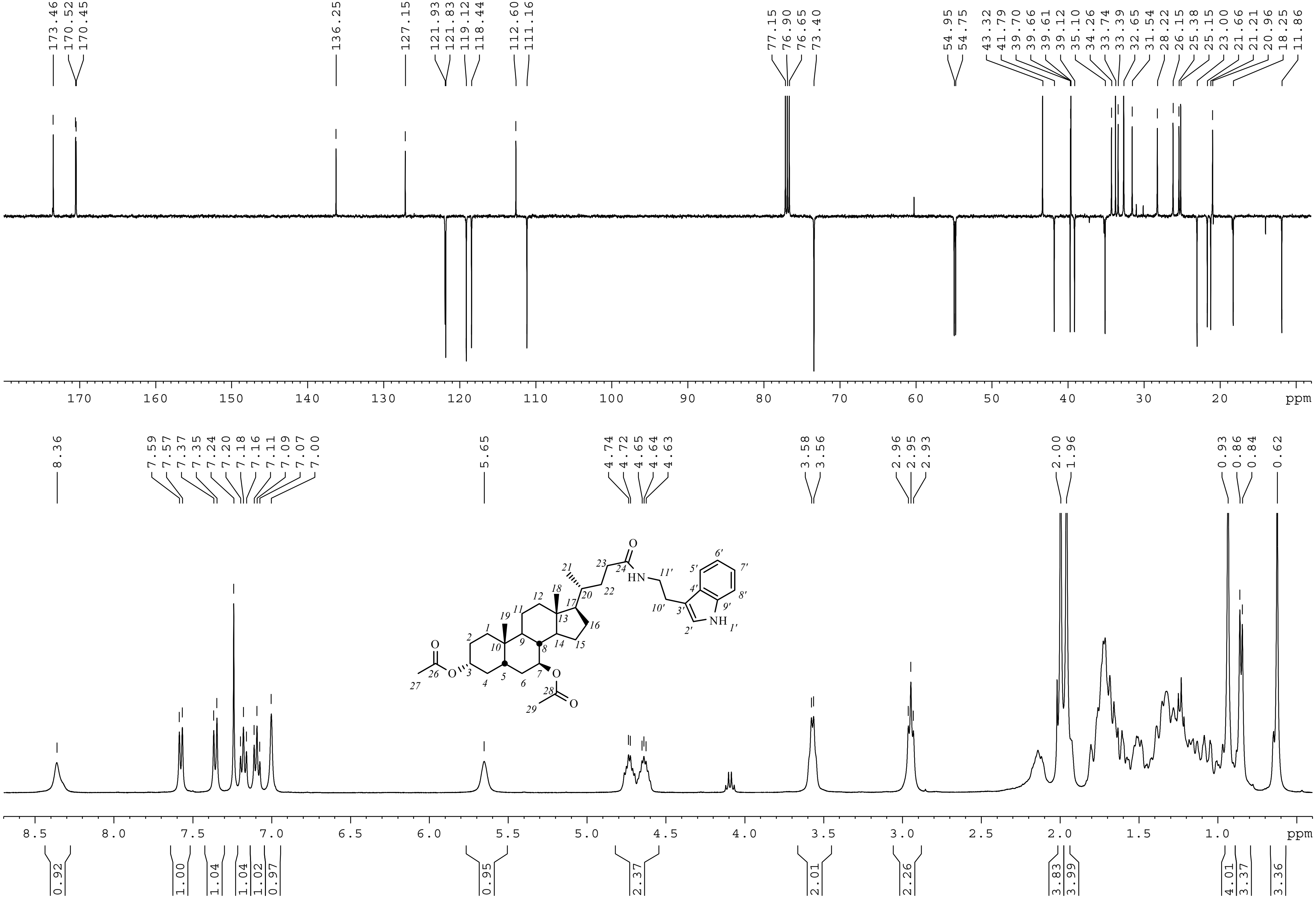

Spectra of Compound **1b**, ^{1}H NMR, 400MHz, $CDCl_3$ (bottom); ^{13}C NMR, BB, 100MHz, $CDCl_3$ (top)

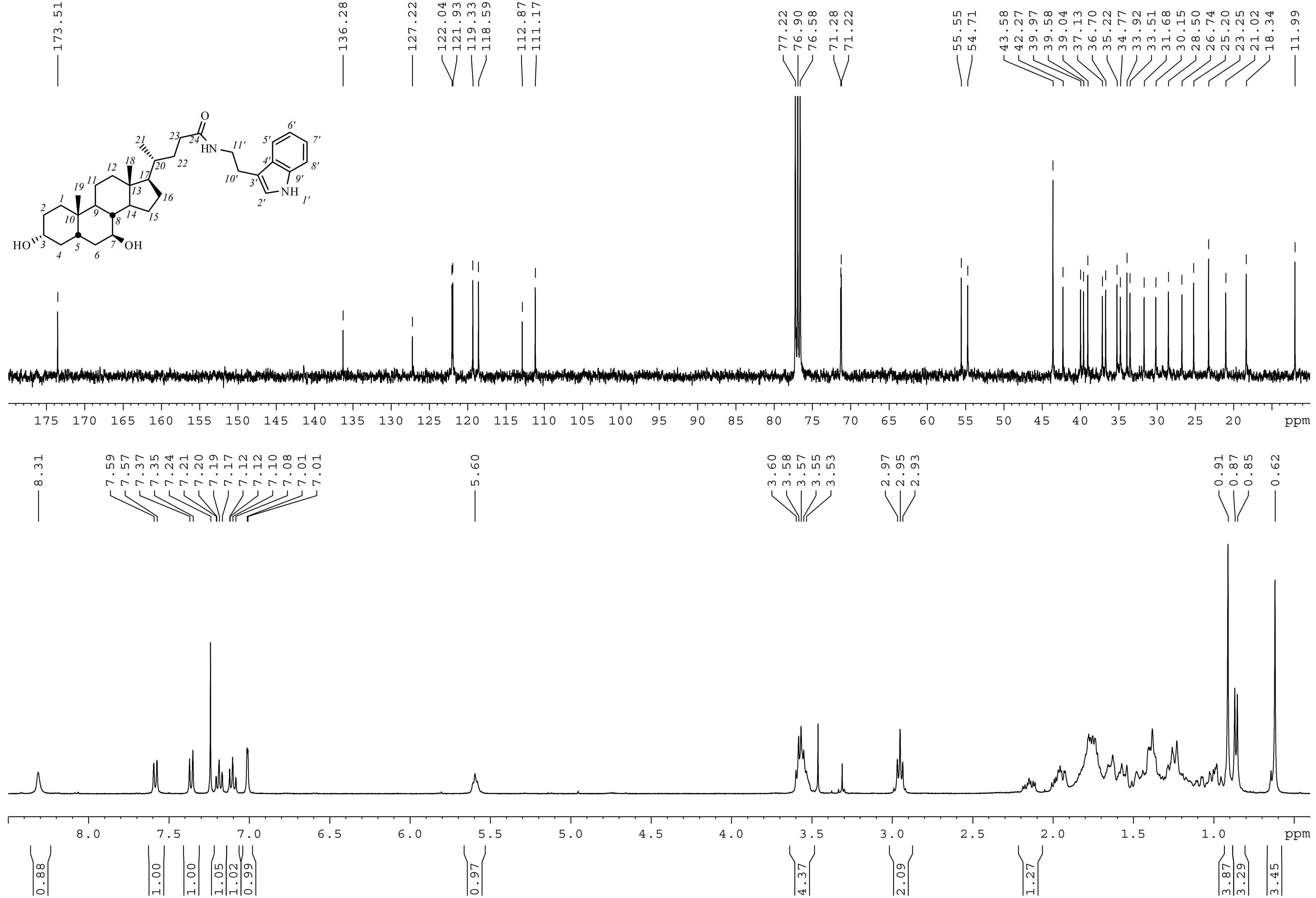

Spectra of Compound **2a**, ^{1}H NMR, 500MHz, $CDCl_3$ (bottom); ^{13}C NMR, JMOD, 125MHz, $CDCl_3$ (top)

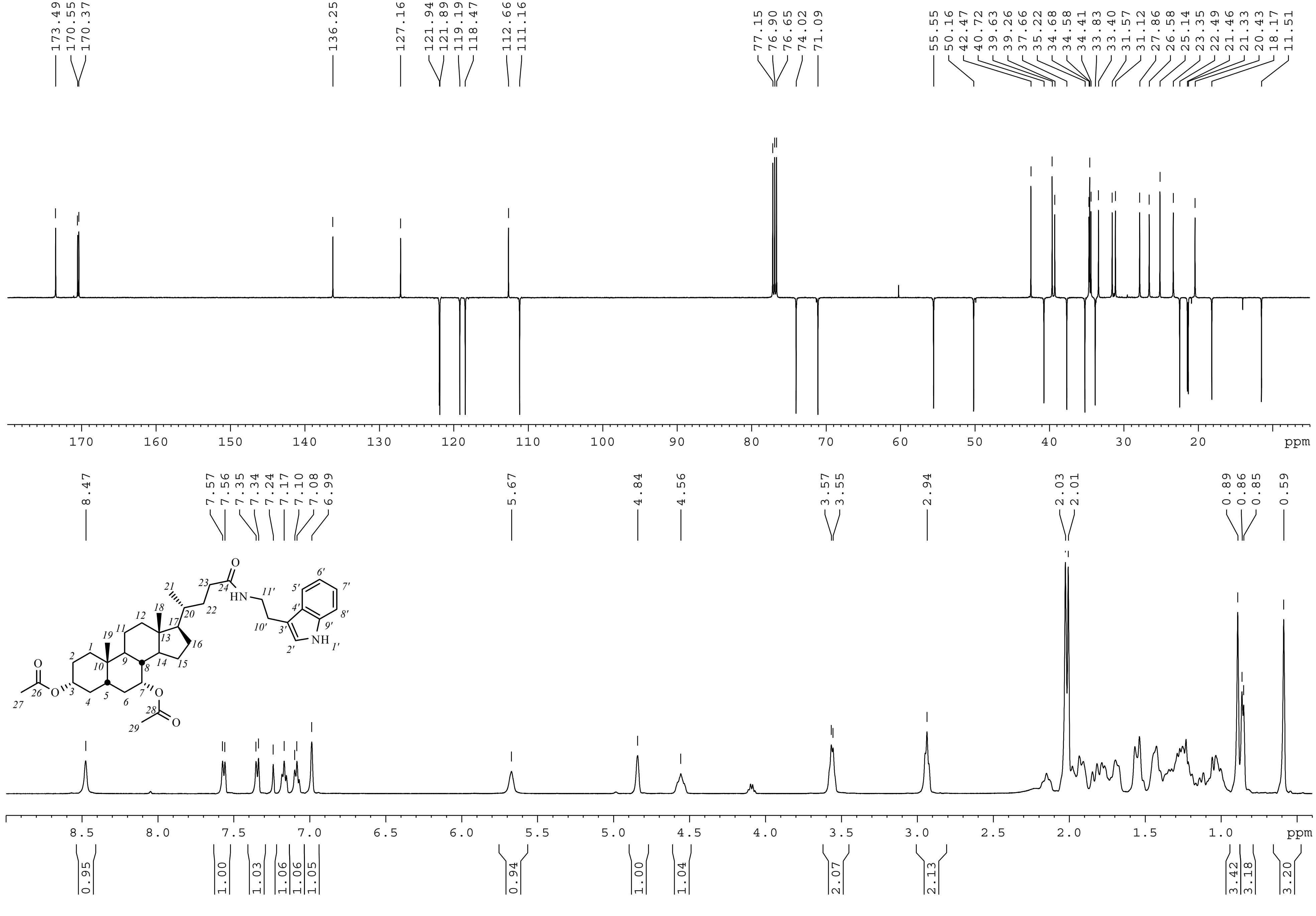

Spectra of Compound **2b**, ^{1}H NMR, 500MHz, $CDCl_3$ (bottom); ^{13}C NMR, JMOD, 125MHz, $CDCl_3$ (top)

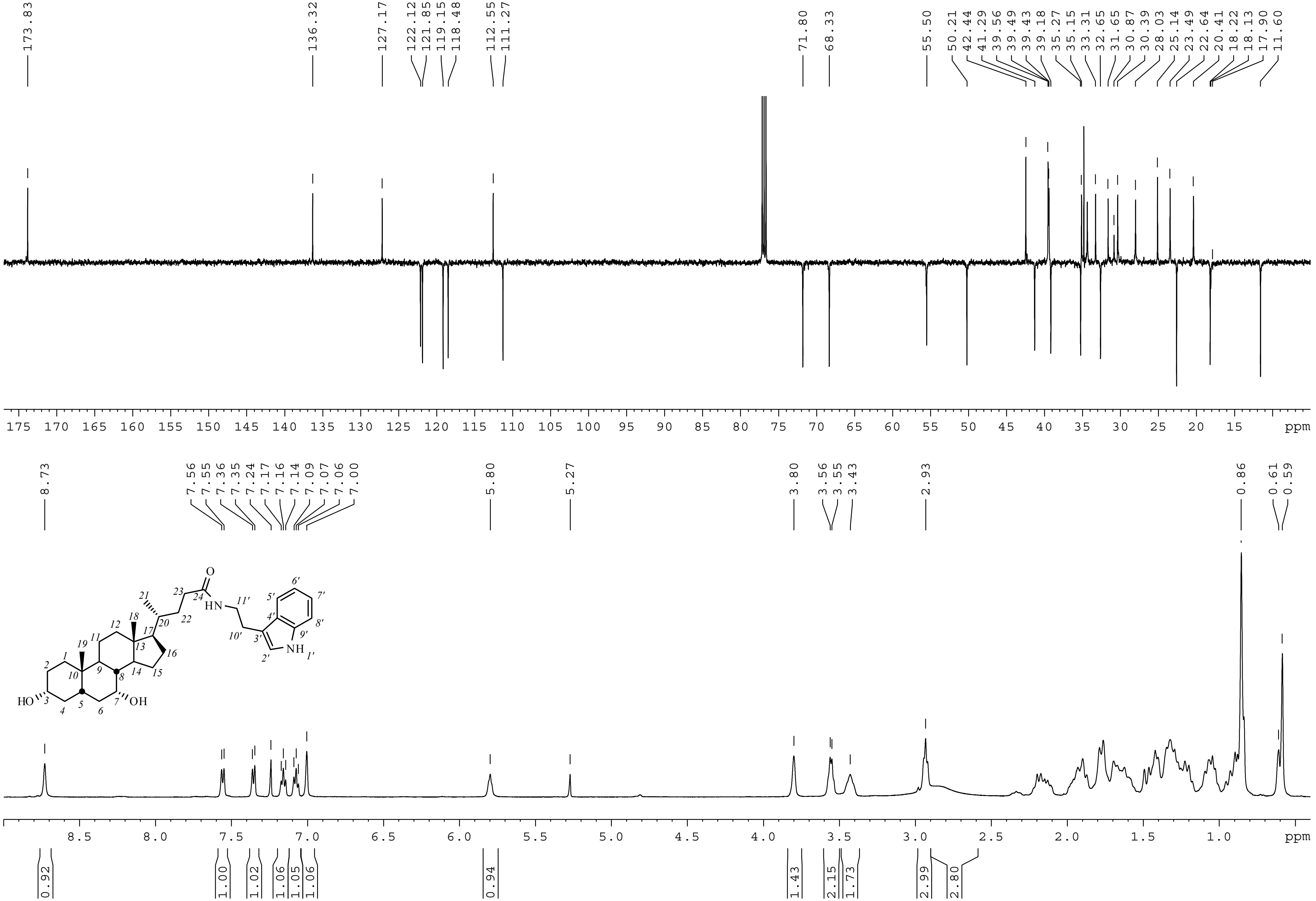

Spectra of Compound **3a**, ^{1}H NMR, 400MHz, $CDCl_3$ (bottom); ^{13}C NMR, JMOD, 100MHz, $CDCl_3$ (top)

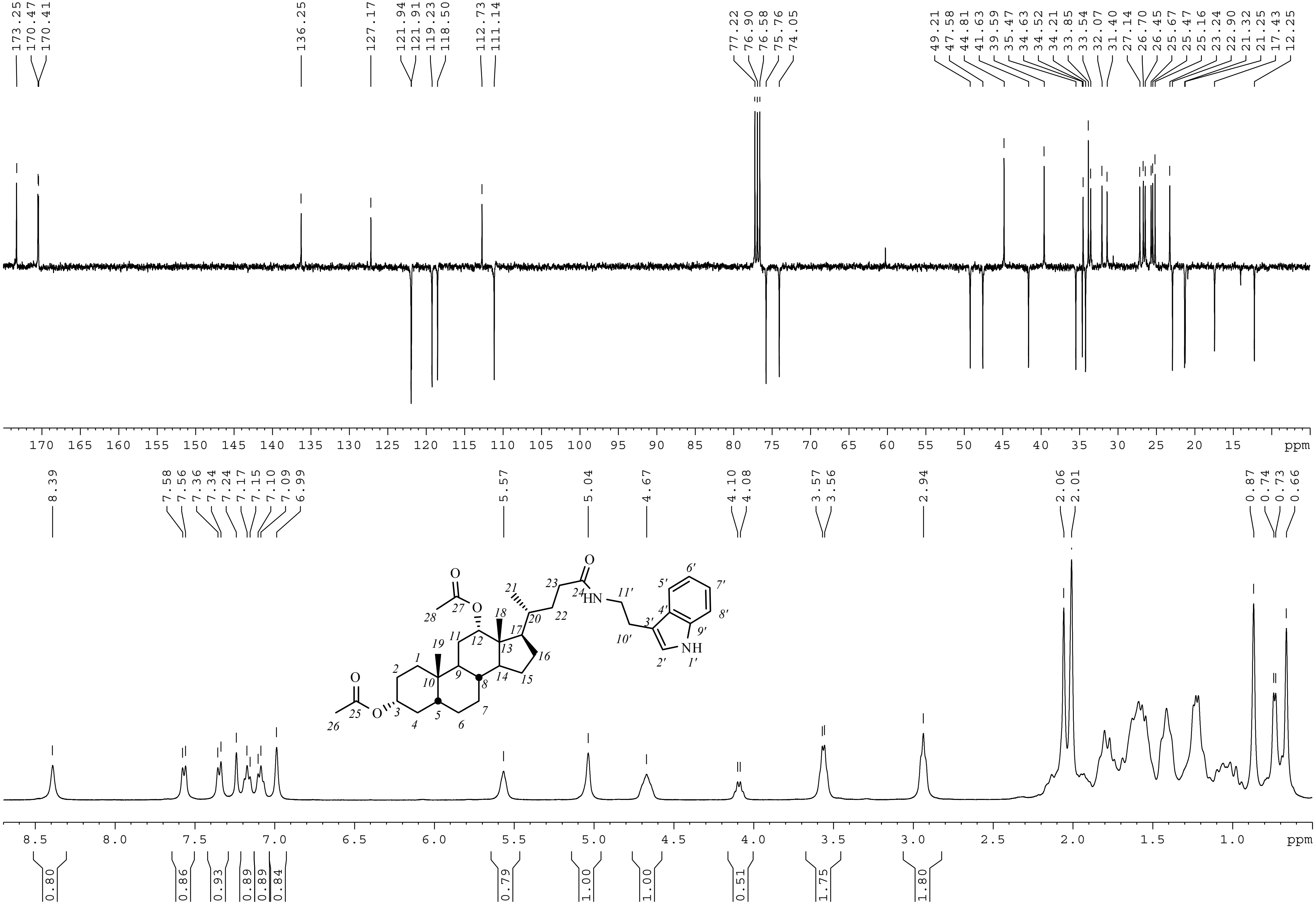

Spectra of Compound **3b**, ^{1}H NMR, 300MHz, $CDCl_3$ (bottom); ^{13}C NMR, JMOD, 75MHz, $CDCl_3$ (top)

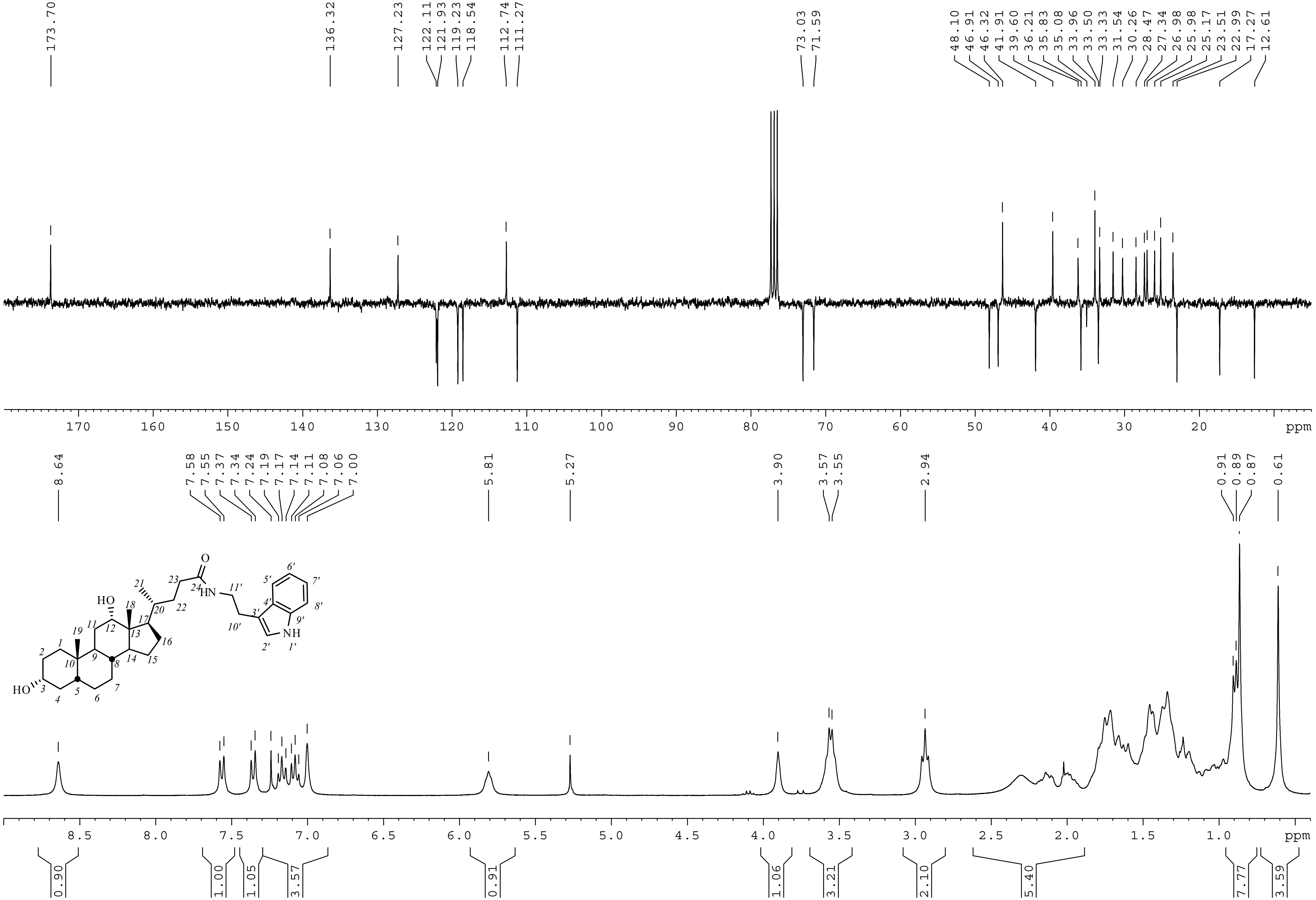

Spectra of Compound **3c**, ^{1}H NMR, 300MHz, $CDCl_3$ (bottom); ^{13}C NMR, JMOD, 75MHz, $CDCl_3$ (top)

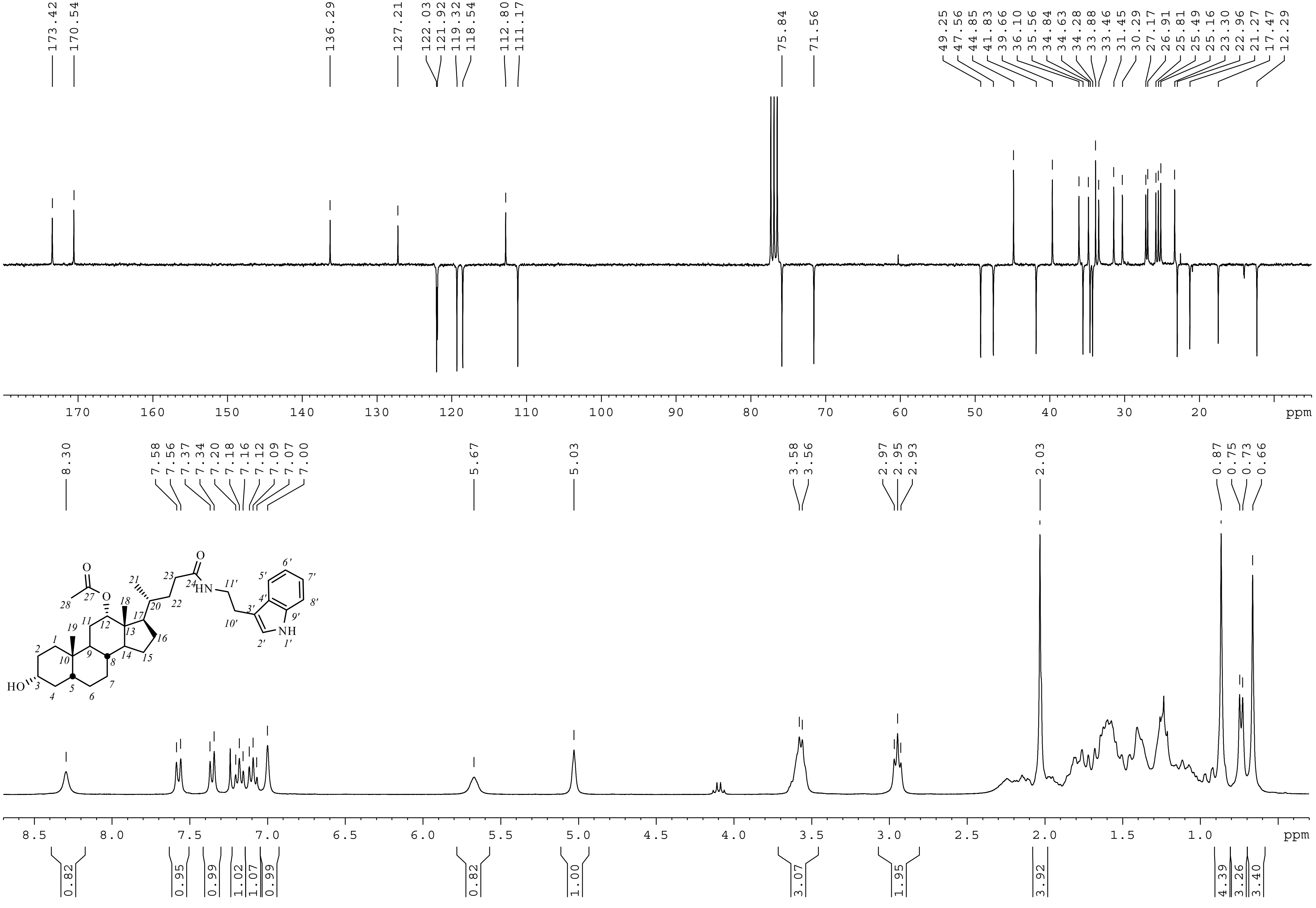

Spectra of Compound **3d**, ^{1}H NMR, 300MHz, $CDCl_3$ (bottom); ^{13}C NMR, JMOD, 75MHz, $CDCl_3$ (top)

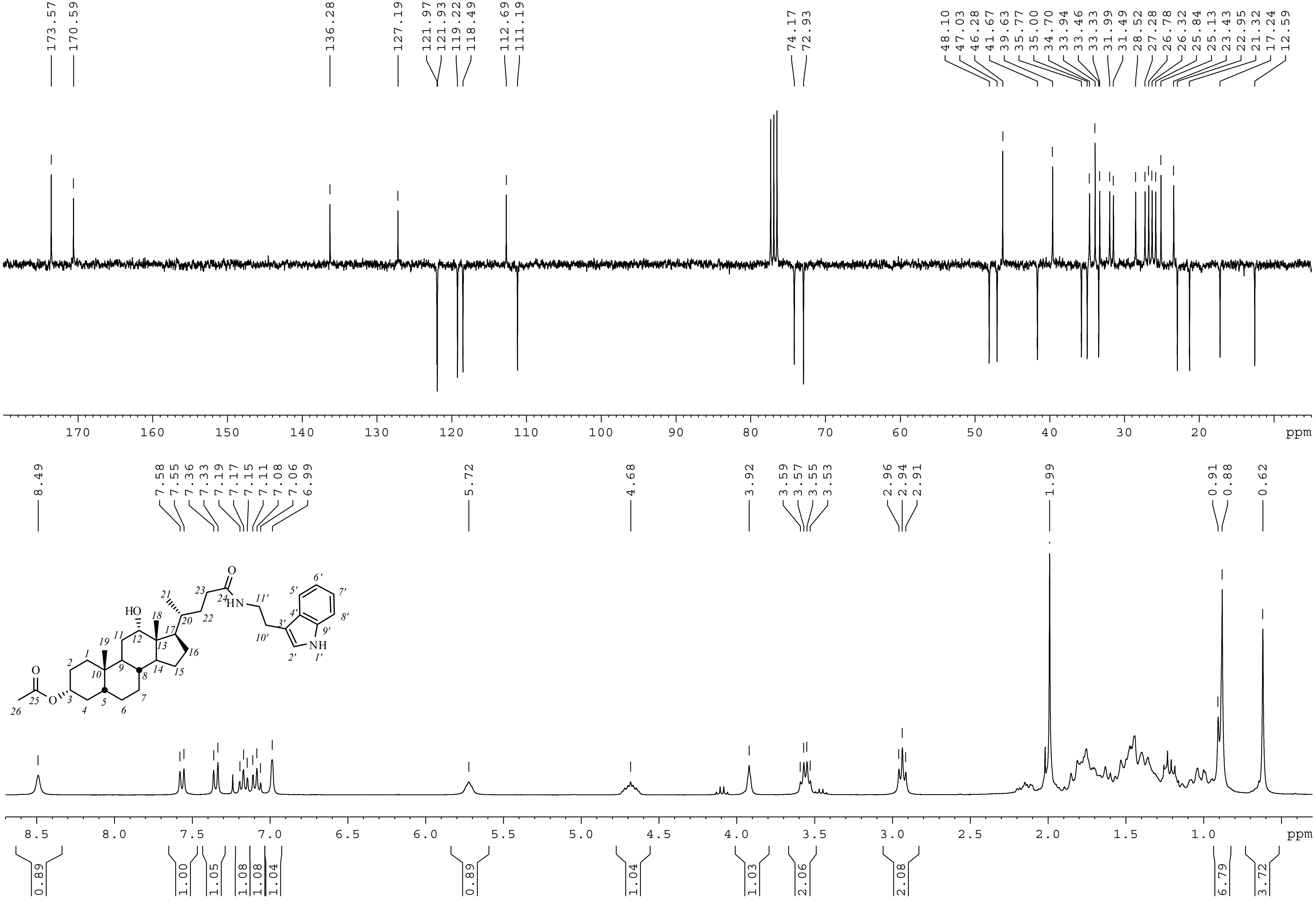

Spectra of Compound **4a**, ^{1}H NMR, 500MHz, $CDCl_3$ (bottom); ^{13}C NMR, JMOD, 125MHz, $CDCl_3$ (top)

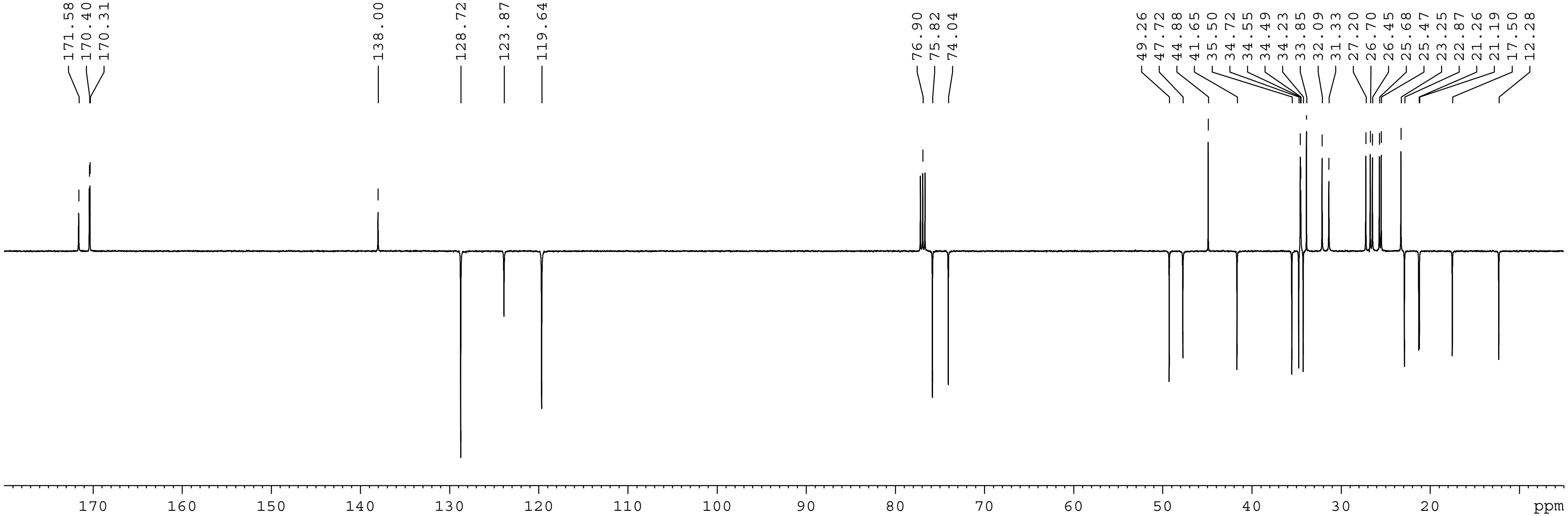

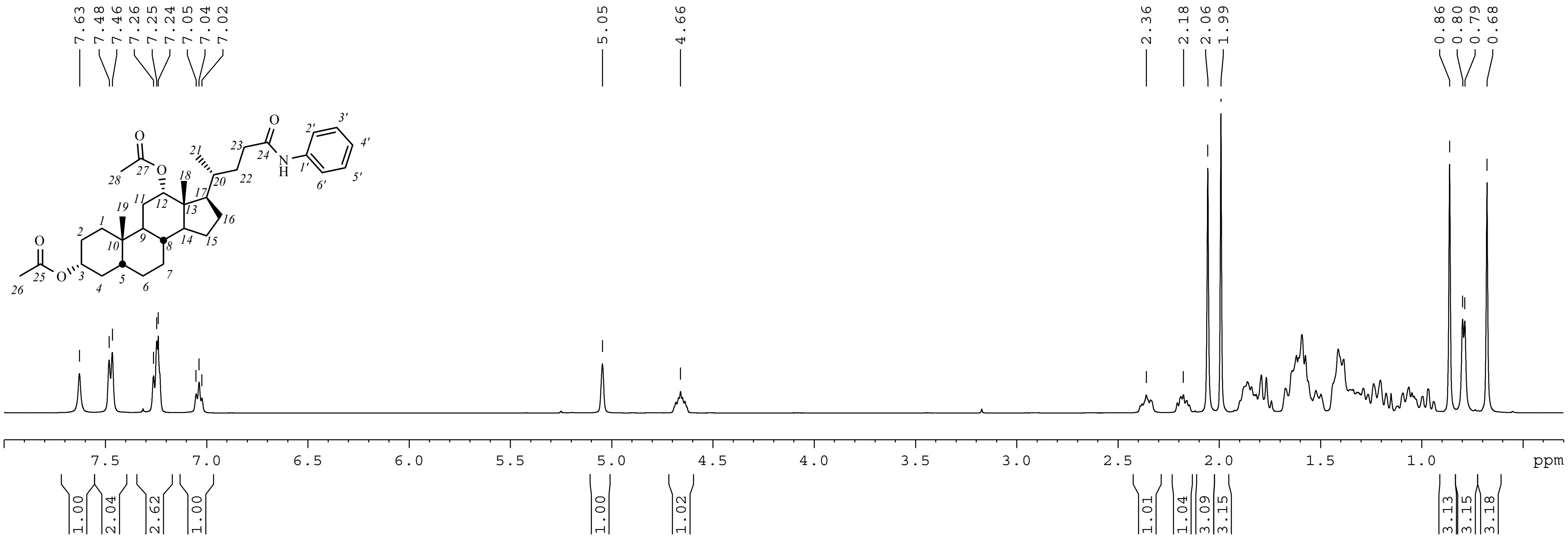

Spectra of Compound **4b**, ^{1}H NMR, 400MHz, $CDCl_3$ (bottom); ^{13}C NMR, JMOD, 100MHz, $CDCl_3$ (top)

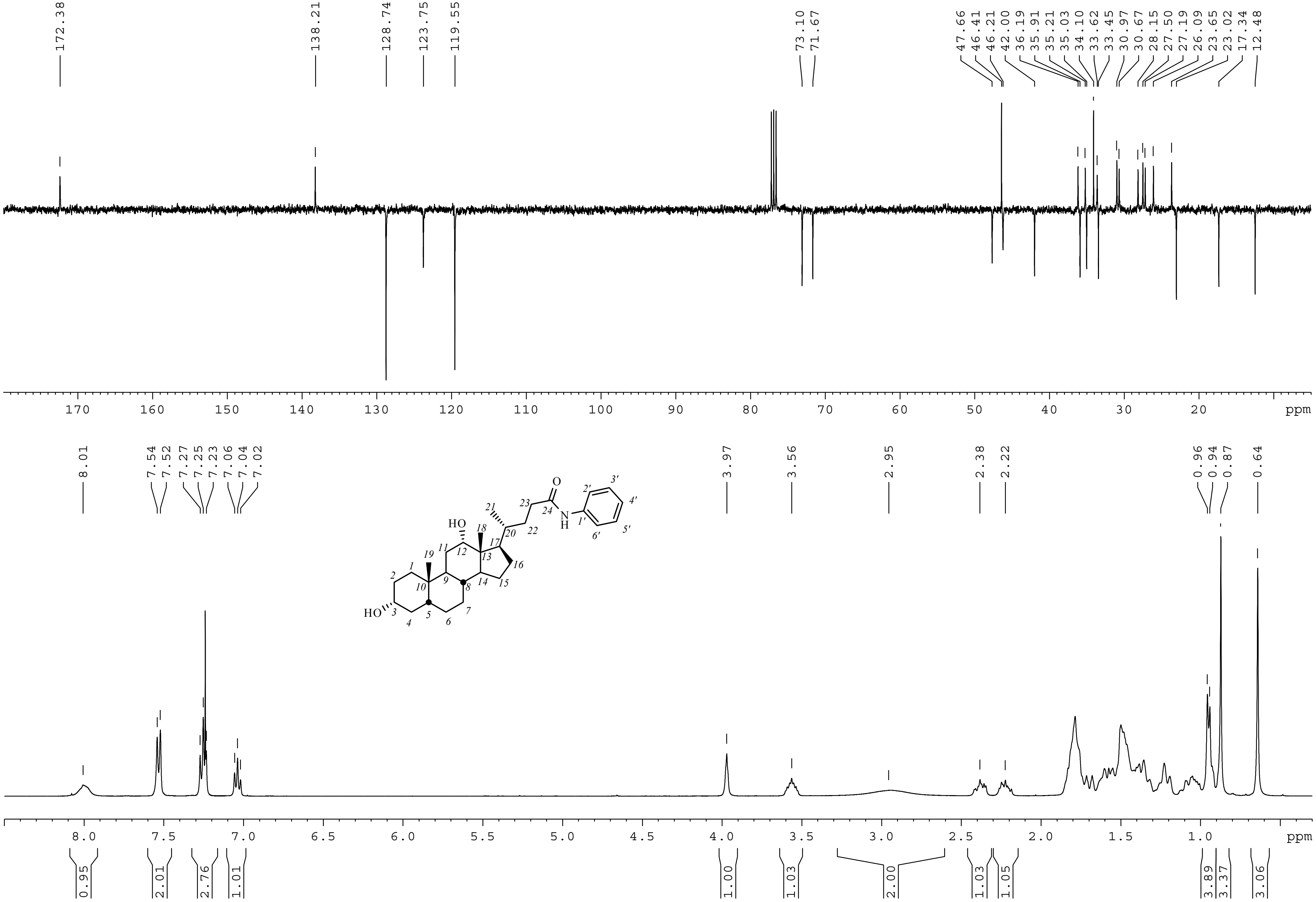

Spectra of Compound **5a**, ^{1}H NMR, 400MHz, $CDCl_3$ (bottom); ^{13}C NMR, JMOD, 100MHz, $CDCl_3$ (top)

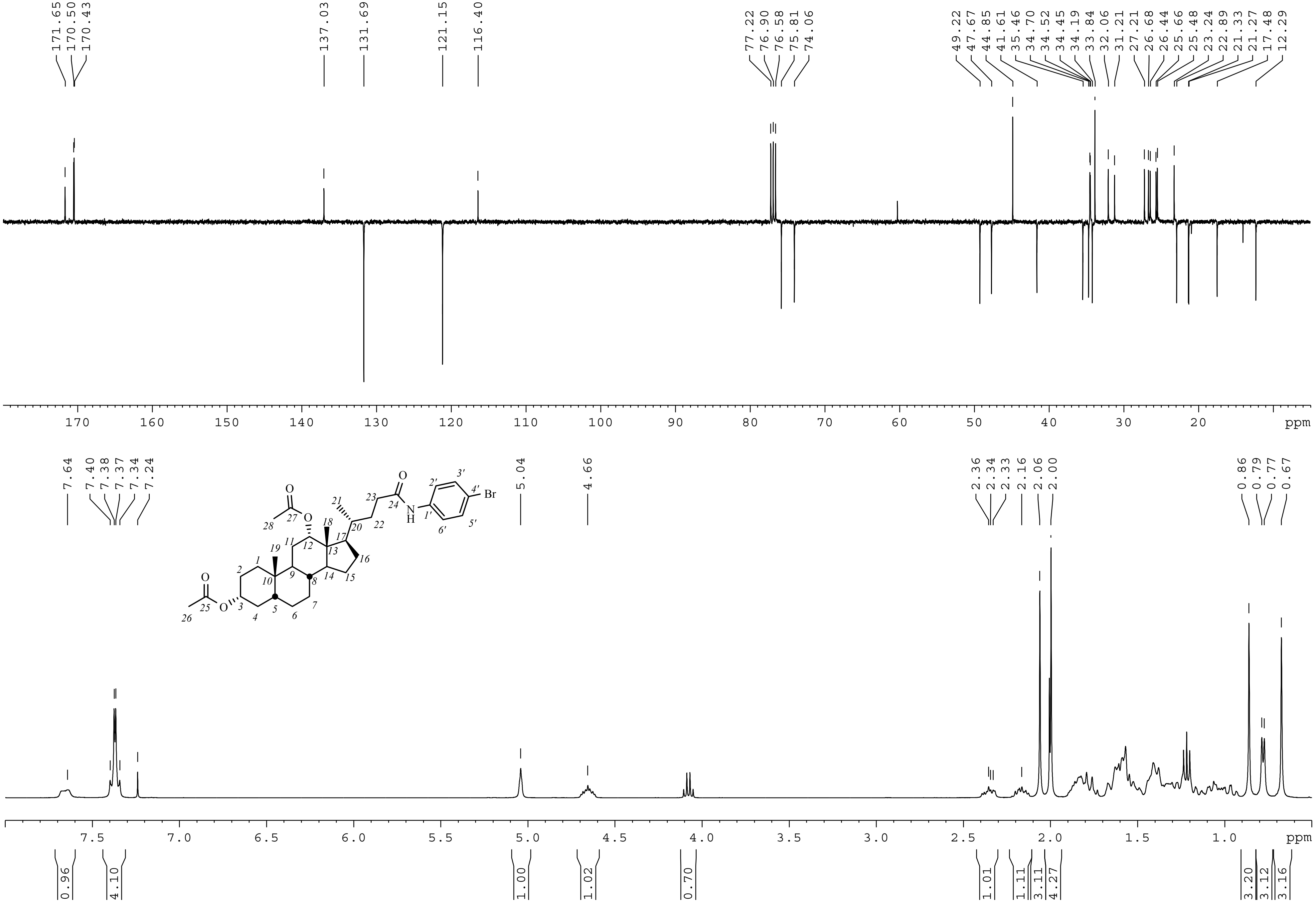

Spectra of Compound **5b**, ^{1}H NMR, 400MHz, $CDCl_3$+CD_3OD (bottom); ^{13}C NMR, JMOD, 100MHz, $CDCl_3$+CD_3OD (top)

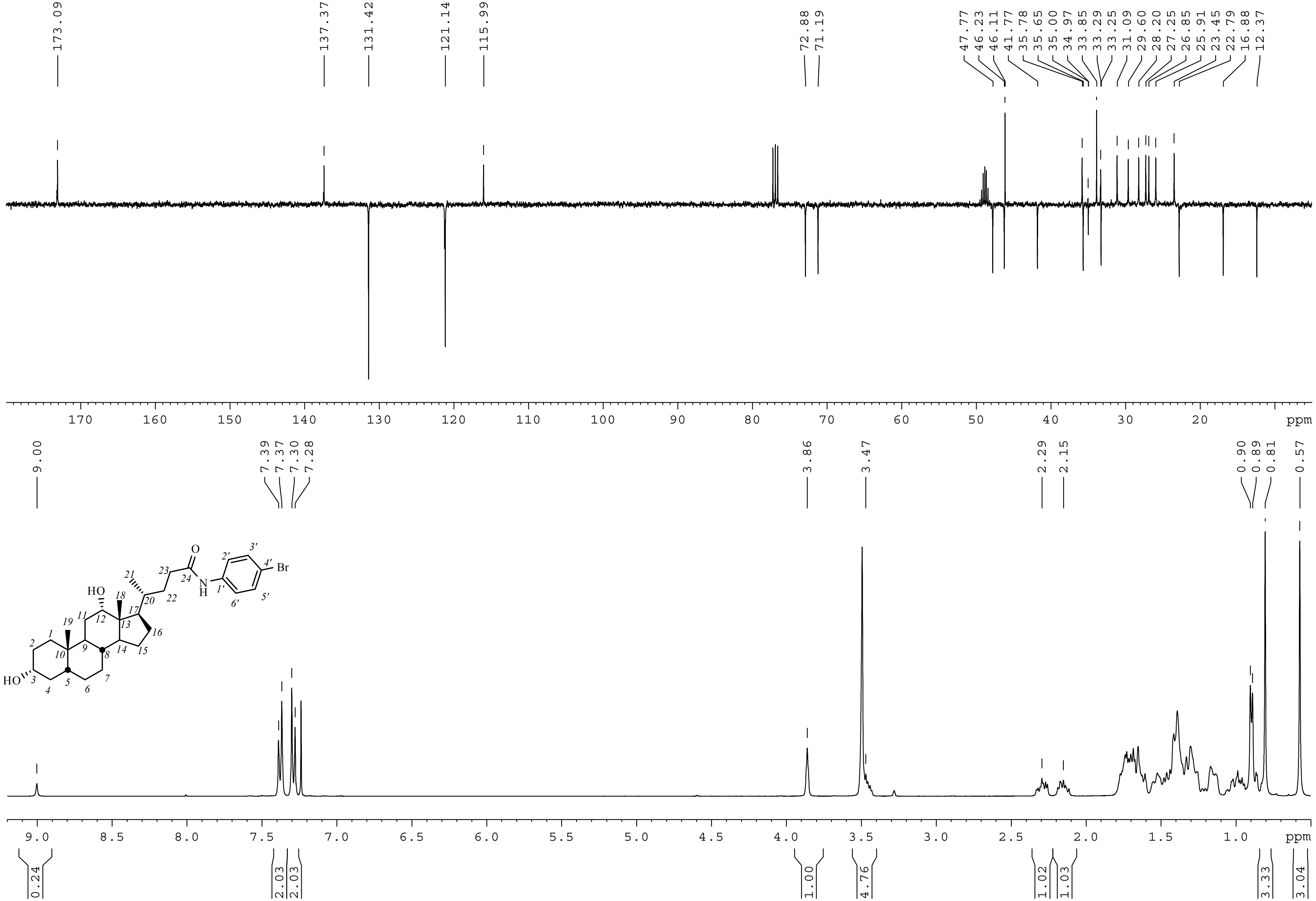

Spectra of Compound **6a**, ^{1}H NMR, 400MHz, $CDCl_3$ (bottom); ^{13}C NMR, JMOD, 100MHz, $CDCl_3$ (top)

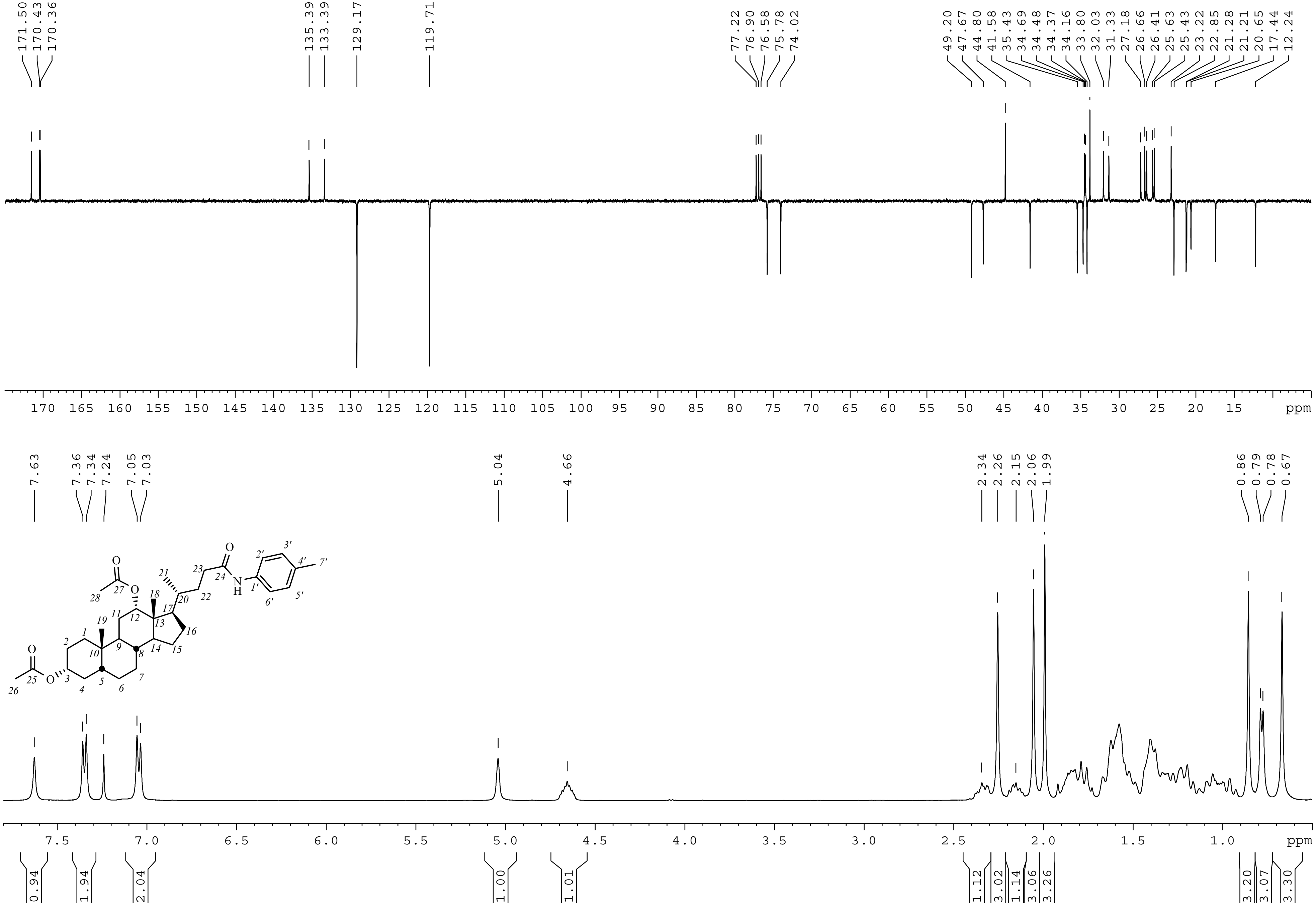

Spectra of Compound **6b**, ^{1}H NMR, 300MHz, $CDCl_3$ (bottom); ^{13}C NMR, JMOD, 75MHz, $CDCl_3$ (top)

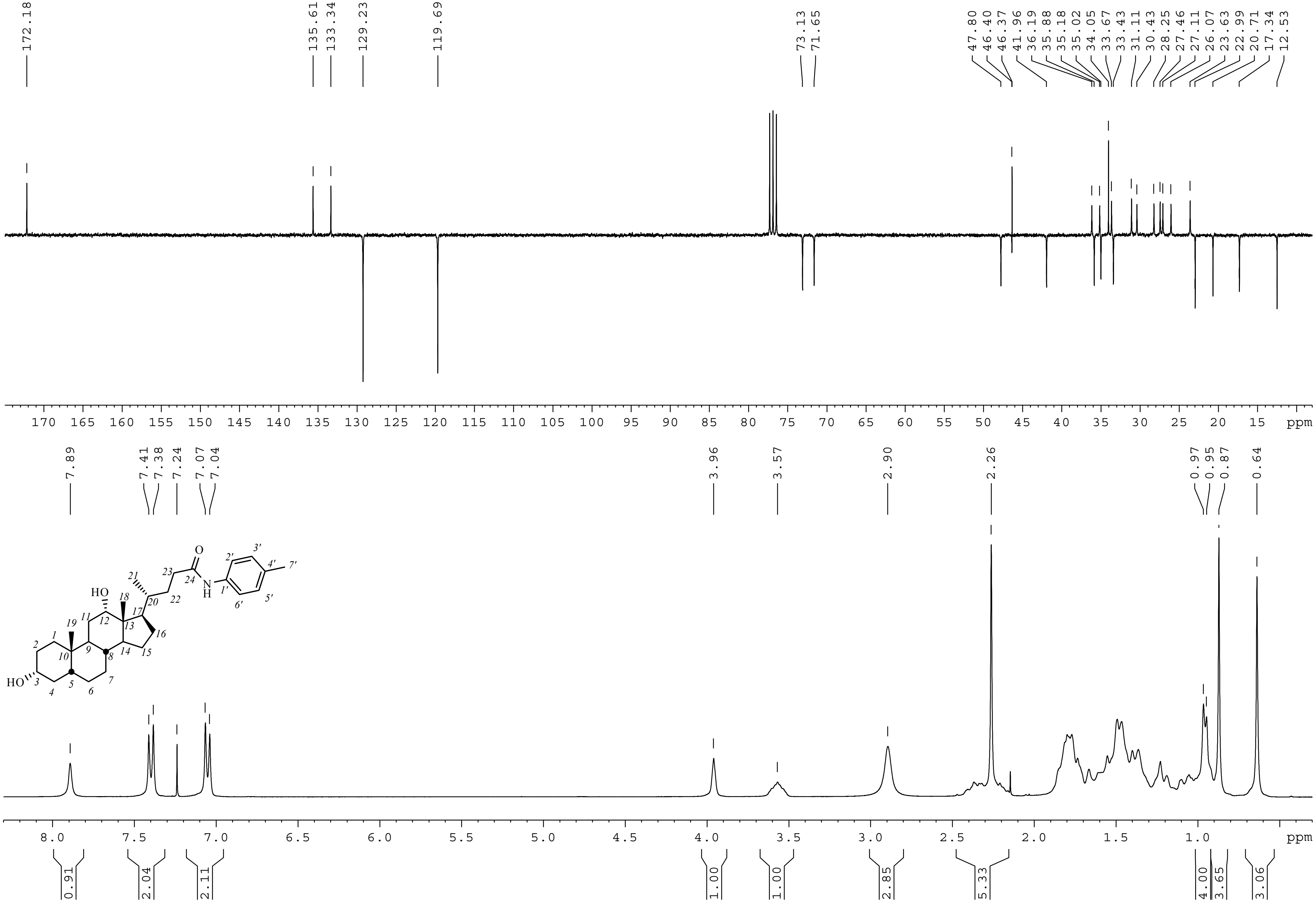

Spectra of Compound **7a**, ^{1}H NMR, 400MHz, CDCl3 (bottom); ^{13}C NMR, JMOD, 100MHz, CDCl3 (top)

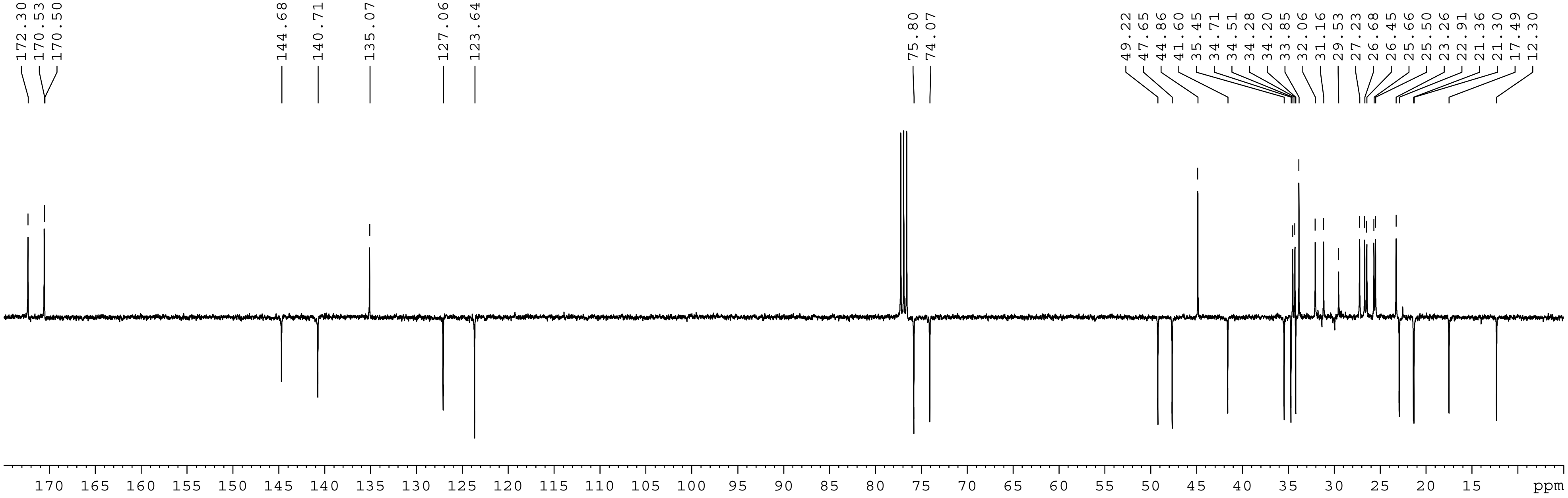

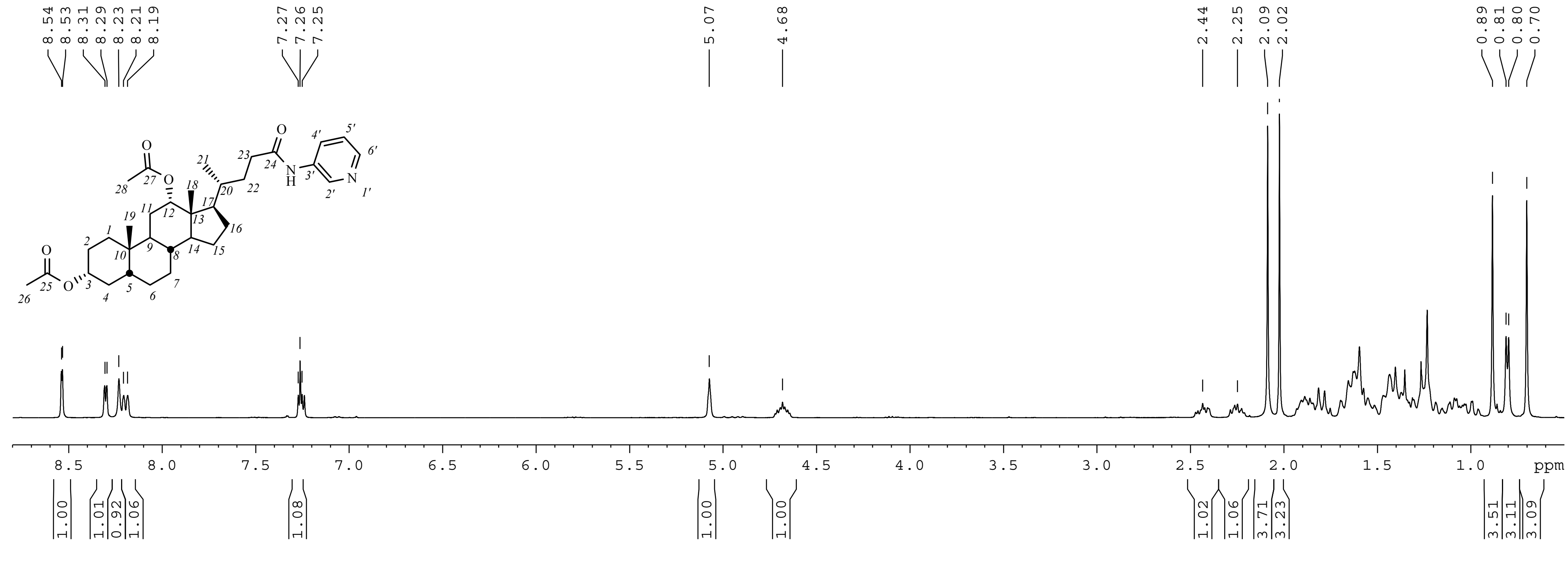

Spectra of Compound **7b**, ^{1}H NMR, 400MHz, $CDCl_3$+CD_3OD (bottom); ^{13}C NMR, JMOD, 100MHz, $CDCl_3$+CD_3OD (top)

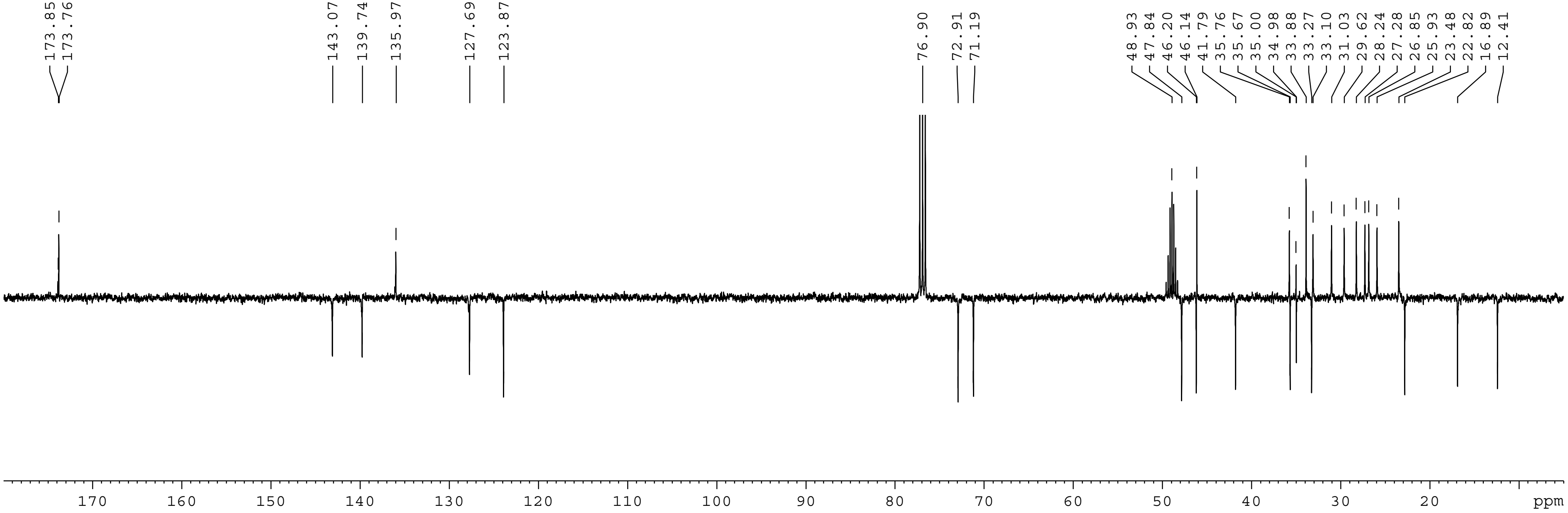

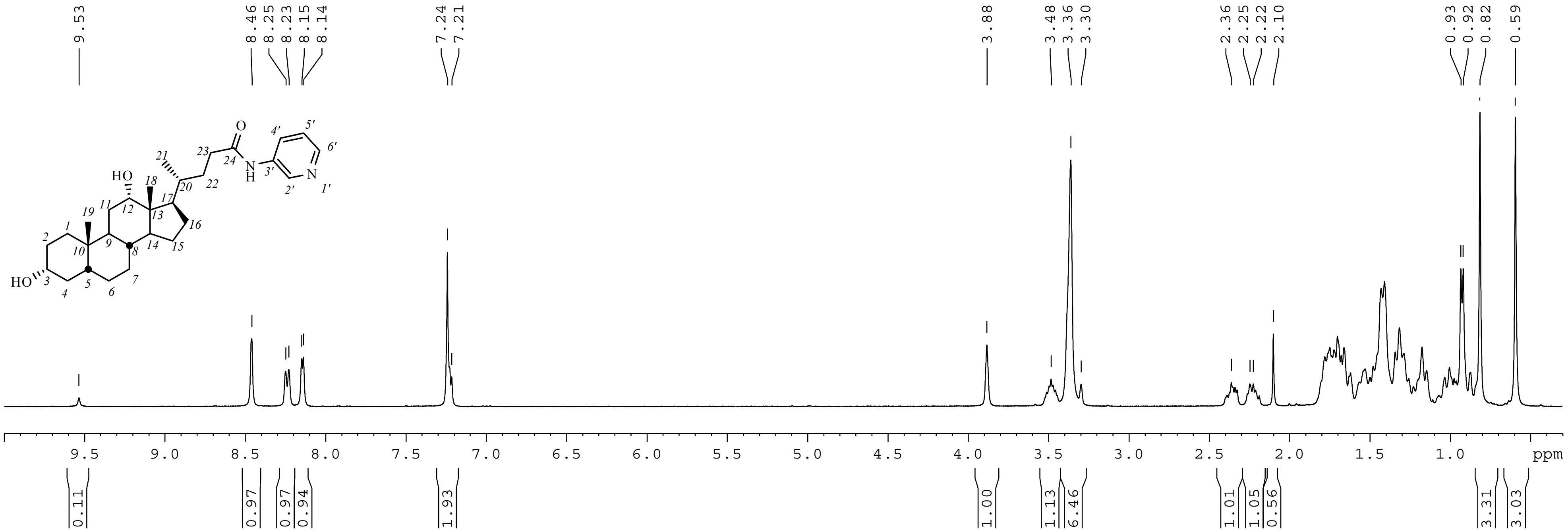

Spectra of Compound **8a**, ^{1}H NMR, 400MHz, $CDCl_3$ (bottom); ^{13}C NMR, JMOD, 100MHz, $CDCl_3$ (top)

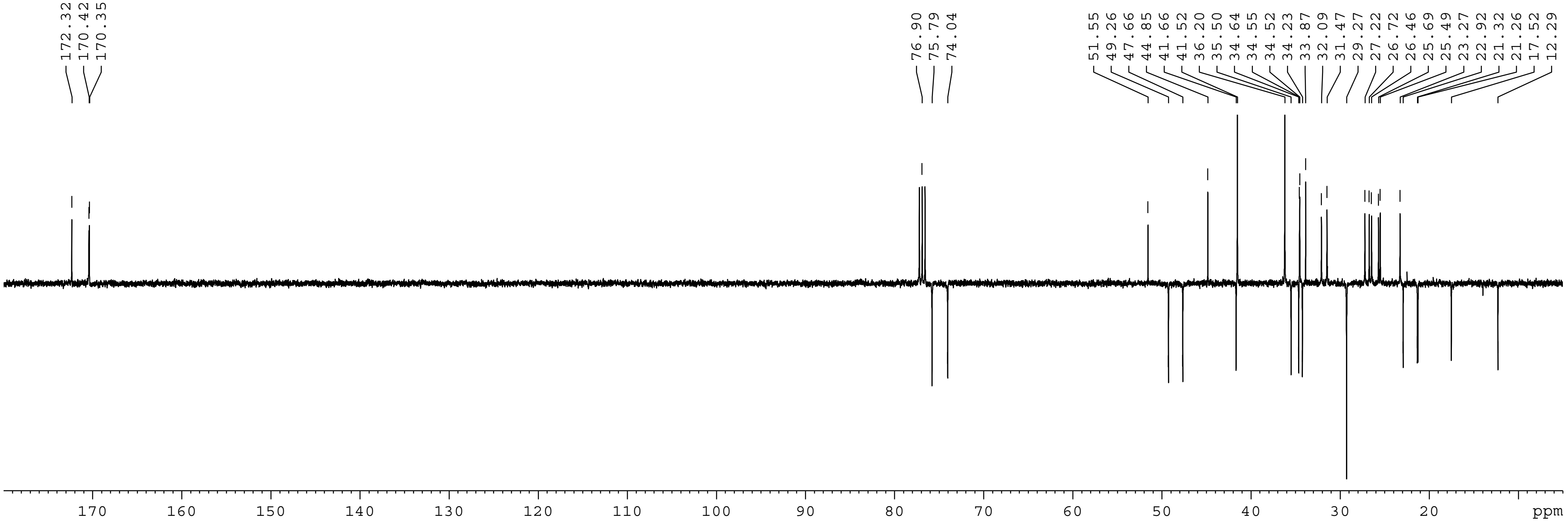

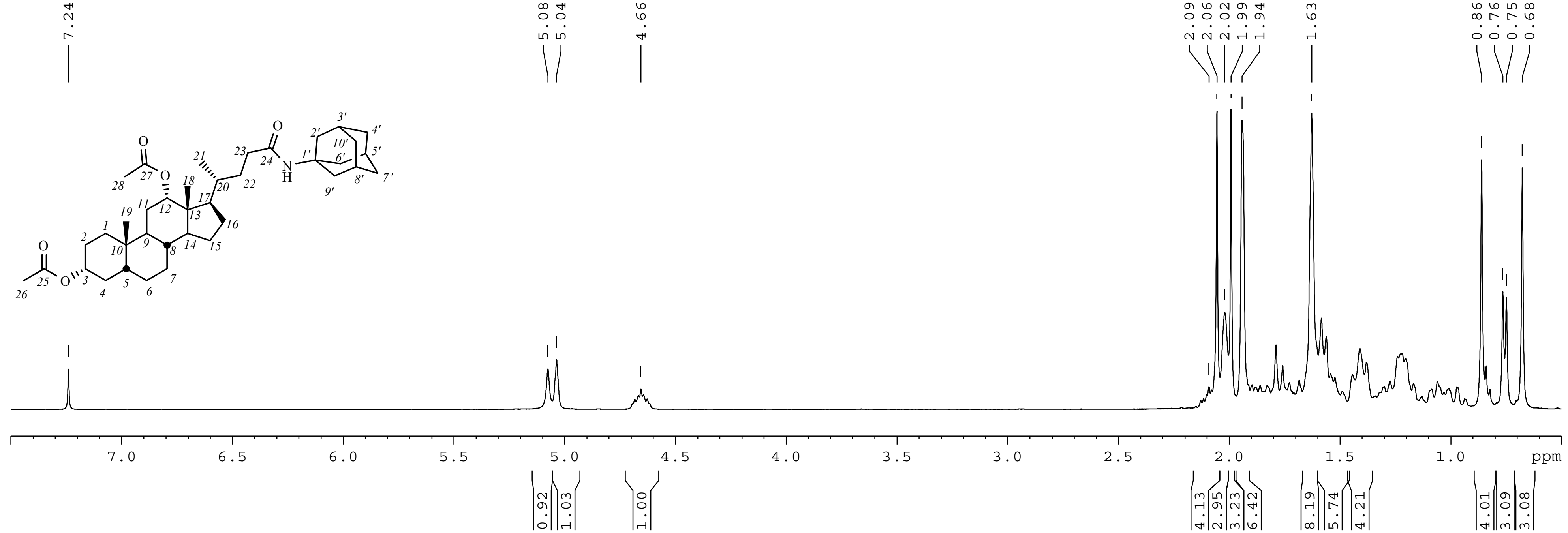

Spectra of Compound **8b**, ^{1}H NMR, 400MHz, $CDCl_3$+CD_3OD (bottom); ^{13}C NMR, JMOD, 100MHz, $CDCl_3$+CD_3OD (top)

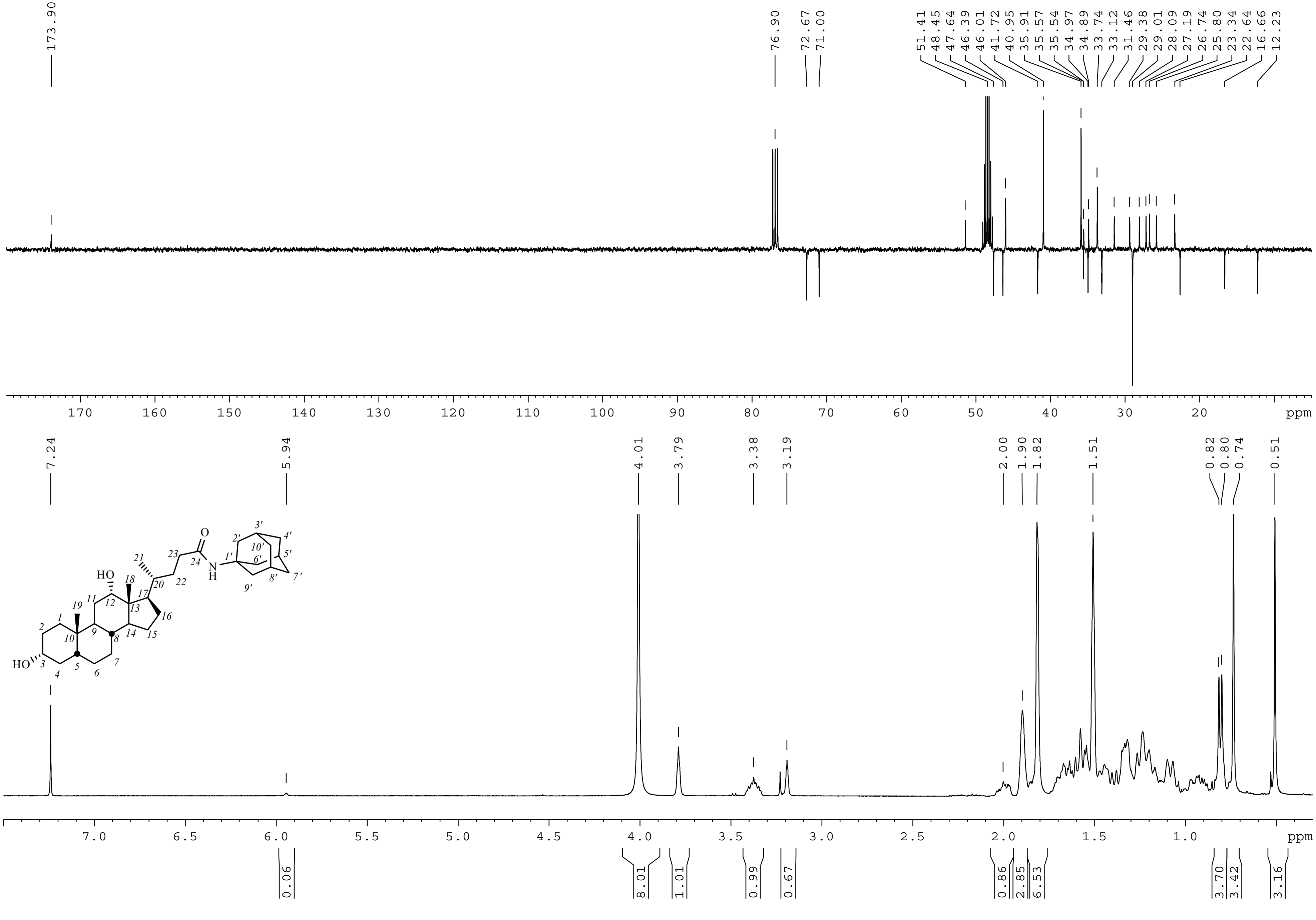

Spectra of Compound **9a**, ^{1}H NMR, 300MHz, $CDCl_3$ (bottom); ^{13}C NMR, JMOD, 75MHz, $CDCl_3$ (top)

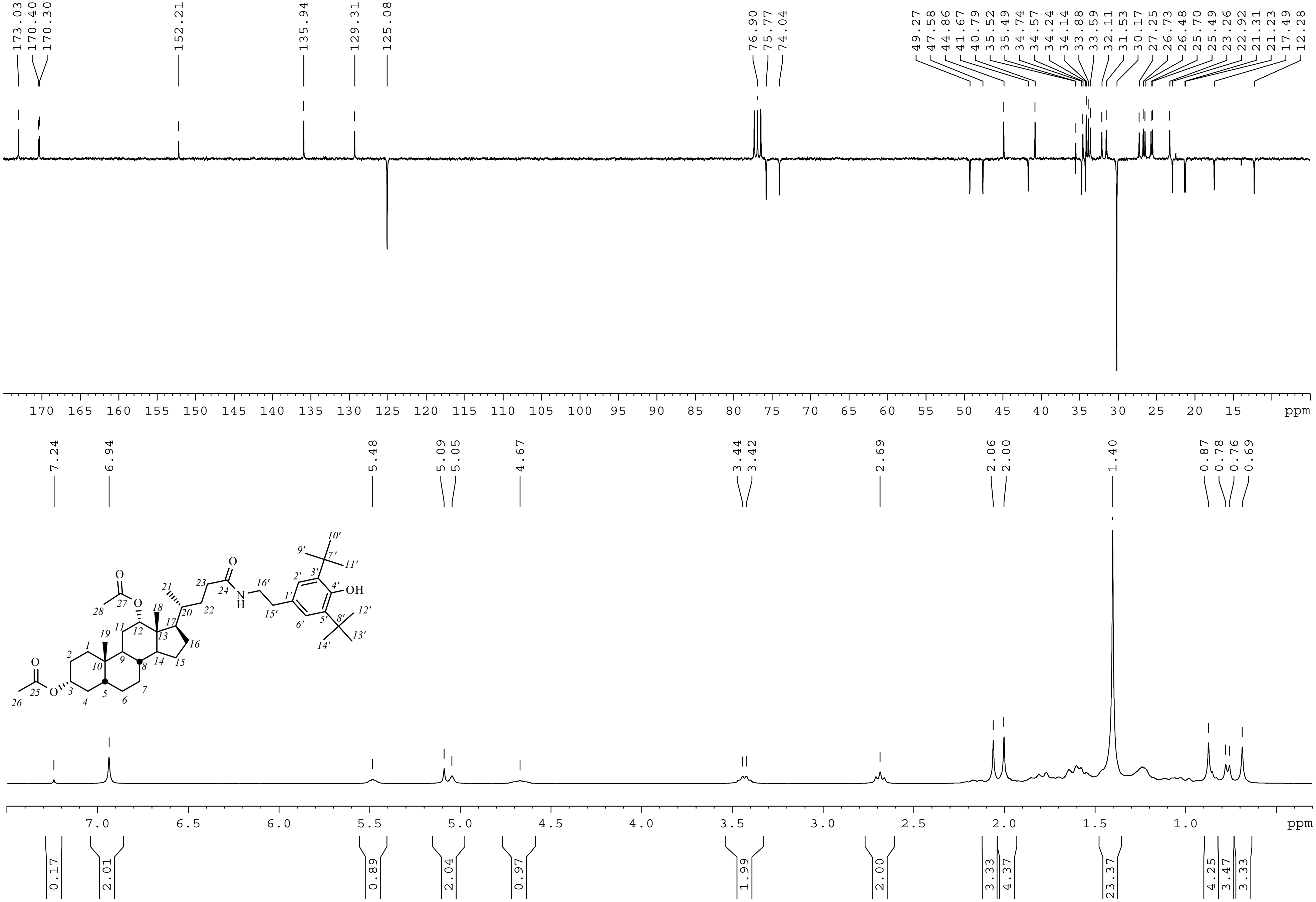

Spectra of Compound **10a**, ^{1}H NMR, 400MHz, $CDCl_3$ (bottom); ^{13}C NMR, JMOD, 100MHz, $CDCl_3$ (top)

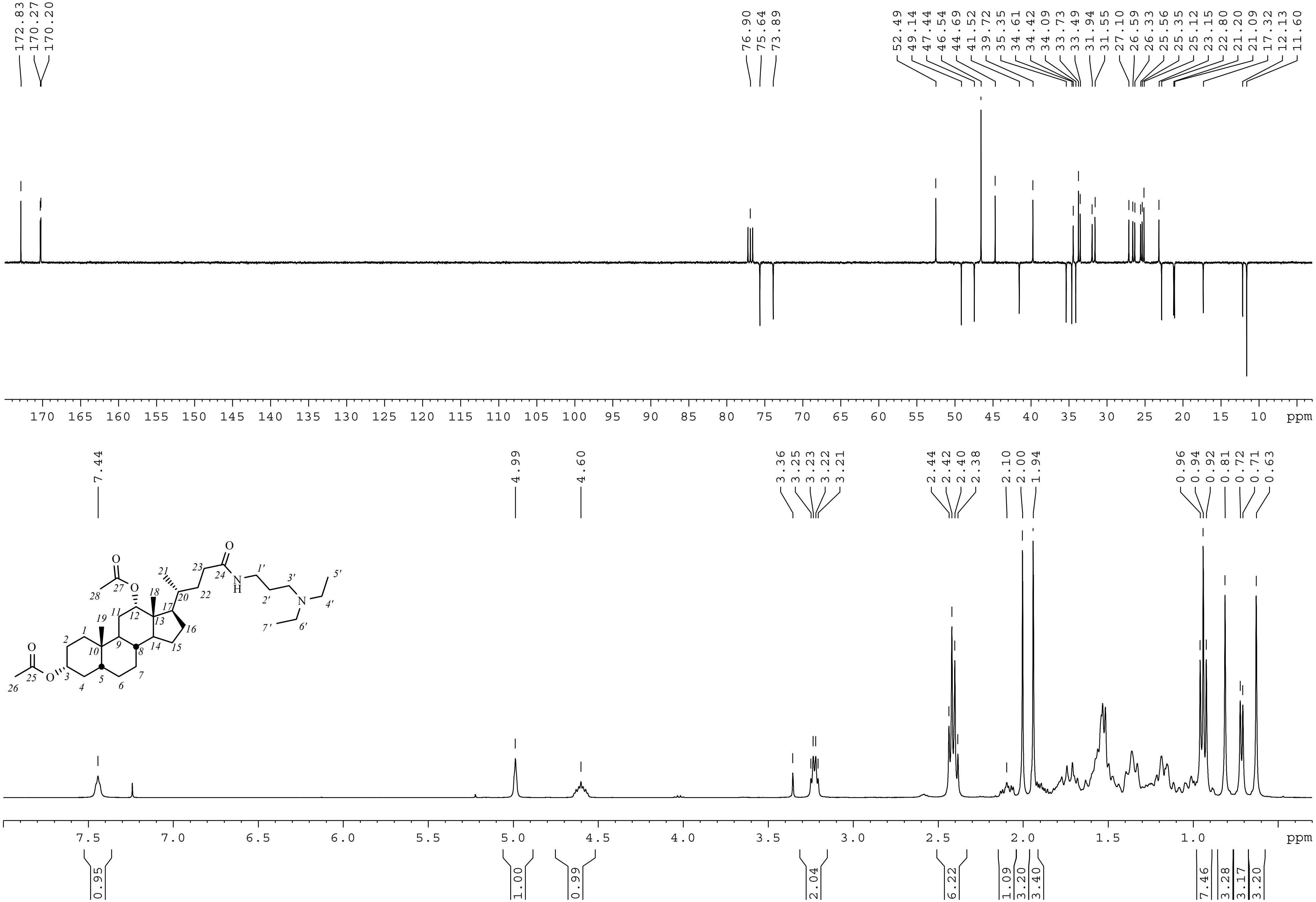

Spectra of Compound **11a**, ^{1}H NMR, 400MHz, $CDCl_3$ (bottom); ^{13}C NMR, JMOD, 100MHz, $CDCl_3$ (top)

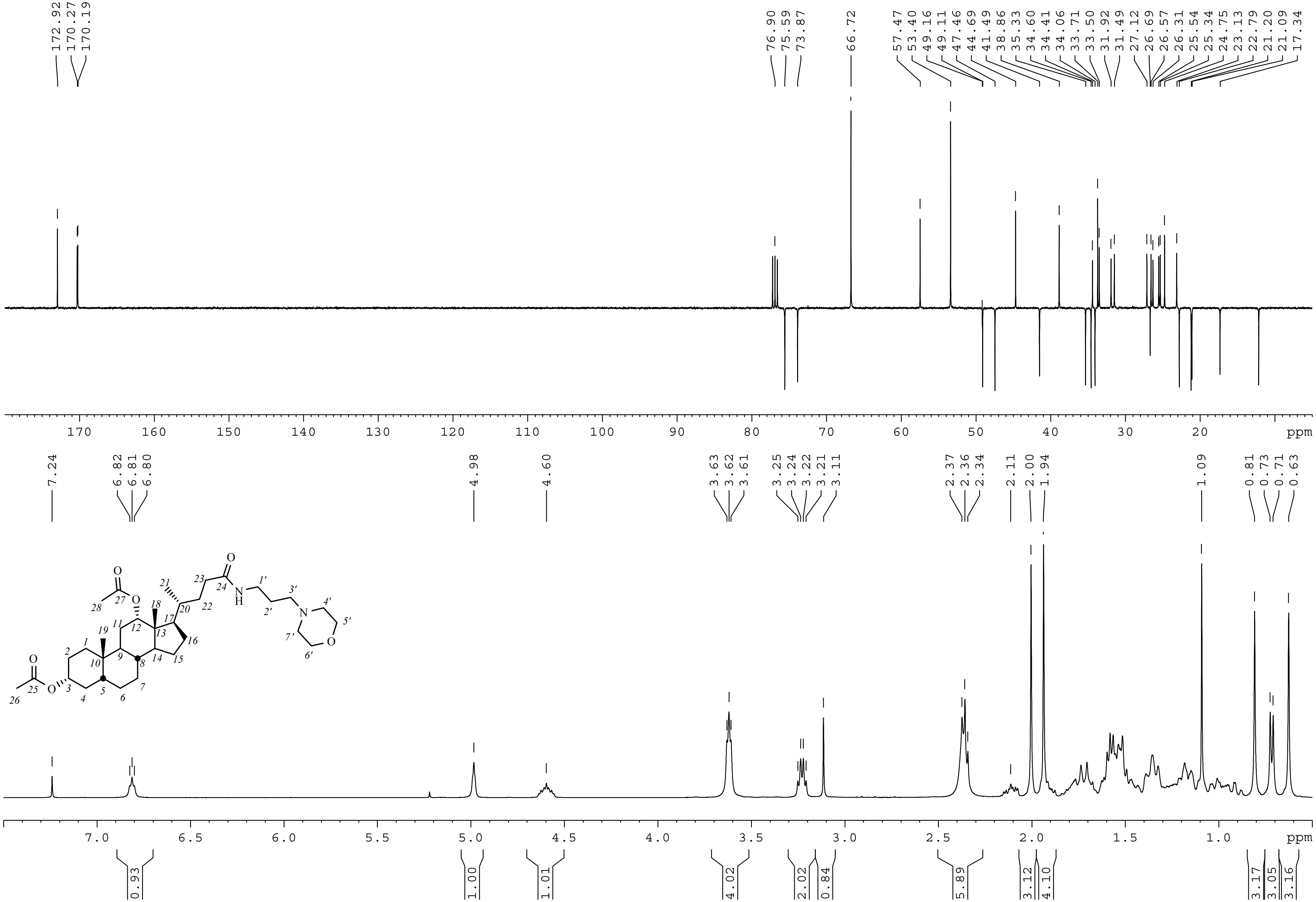

Supplement: Supplementary file 1 [file molecules-23-00679-s001.pdf]
